# Dynamic upconversion multicolour editing enabled by molecule-assisted opto-electrochemical modulation

**DOI:** 10.1038/s41467-021-22387-7

**Published:** 2021-04-01

**Authors:** Yiming Wu, Jiahui Xu, Xian Qin, Jun Xu, Xiaogang Liu

**Affiliations:** 1 Department of Chemistry, National University of Singapore, Singapore, Singapore; 2 Center for Functional Materials, National University of Singapore Suzhou Research Institute, Suzhou, China; 3 Joint School of National University of Singapore and Tianjin University, International Campus of Tianjin University, Fuzhou, China; 4 Institute of Materials Research and Engineering, A*STAR, Singapore, Singapore

**Keywords:** Nanophotonics and plasmonics, Molecular electronics

## Abstract

Controlling nonlinear optical signals electrically offers many opportunities for technological developments. Lanthanide-activated nanoparticles have recently emerged as leading platforms for nonlinear upconversion of infra-red excitation within nanometric volumes. However, manipulation of upconversion emission is restricted to varying percentages of component materials, nanocrystal structure, and optical pumping conditions. Here, we report temporal modulation of anti-Stokes luminescence by coupling upconversion nanoparticles with an electrochemically responsive molecule. By electrically tailoring orbital energy levels of the molecules anchored on nanoparticle surfaces, we demonstrate reversible control of molecular absorption, resulting in dynamic colour editing of anti-Stokes luminescence at single-particle resolution. Moreover, we show that a programmable logic gate array based on opto-electrochemical modulation can be constructed to convert information-encrypted electrical signals into visible patterns with millisecond photonic readout. These findings offer insights into precise control of anti-Stokes luminescence, while enabling a host of applications from low-threshold infrared logic switches to multichannel, high-fidelity photonic circuits.

## Abstract (web summary)

Though upconversion nanoparticles (UCNPs) are attractive for infrared detecting and modulating photonic devices, devising strategies to electrically manipulate upconversion emission remain a challenge. Here, the authors report molecule-assisted opto-electrochemical modulation of UCNP luminescence.

## Introduction

Nonlinear photonic devices capable of detecting and modulating infrared signals as a communication medium are essential for technological developments in thermography, night vision, medical diagnosis, information encryption, and optical communication^1–6^. Such optoelectronic applications can be accomplished by using nonlinear nanocrystals that enable frequency conversion of invisible infrared radiation to visible luminescence. The ability to dynamically control optical functionalities of the upconversion medium by modulating external stimuli, such as temperature, pressure, magnetic or electric field, will facilitate development of multifunctional, nonlinear optoelectronic devices^7–9^. In particular, there is strong demand for electrical control over optical properties of upconversion nanomaterials, as such control will not only allow monolithic integration of photonic elements with optoelectronic functionalities but will also lead to many intriguing phenomena in nonlinear regimes^10–12^.

Compared with conventional nonlinear nanocrystals, lanthanide-activated upconversion nanoparticles (UCNPs) allow more-efficient frequency upconversion owing to abundant, physically existing energy states. UCNPs also exhibit excellent photostability, large anti-stokes shift, low pumping threshold, and broadly tunable multicolour emission^13,14^. Temporal modulation of upconversion emission can be realised by varying excitation wavelengths or by adjusting excitation power densities^15–19^. These methods, however, require multiple excitation sources and stringent control over crystalline phase, chemical composition, and surface ligands. Variations in the thermal field also allow reversible luminescence modulation of UCNPs, though these are impractical for device configuration^20,21^. External stimulus-responsive hosts, such as liquid crystal polymer networks, ferroelectric or optomagnetic materials, have been studied to manipulate emission dynamically under external electrical or magnetic stimulation^22–25^. However, high phonon energy and low upconversion efficiency of these systems, as well as a limited range of emission colours, hinder their practical utility for multicolour switching. Despite enormous efforts, implementation of reversible, dynamic, full-colour emission modulation of UCNPs through electric field stimulation has not been achieved, largely owing to the non-conducting nature of conventional lanthanide-doped host materials and shielding effects of 4f electrons against external electrostatic perturbation.

**Fig. 1:**
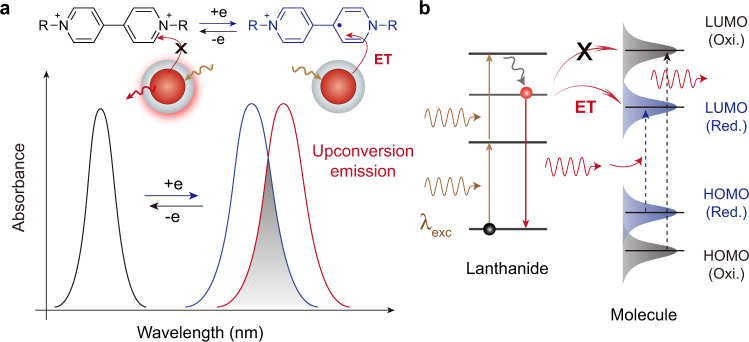
Molecular-assisted surface electrochemical tuning (MASET). **a** Schematic illustration of electrochemically controlled energy transfer (ET) from UCNPs to viologen molecules (R represents a functional group). An applied electric field induces electrochemical conversion of viologen molecules. For a given set of nanocrystals and viologen molecules, no energy transfer occurs upon NIR laser excitation (*λ*_exc_), owing to lack of spectral overlap between nanocrystal emission and dye absorption. Applying an external electric field drives the oxidised state (Oxi.) of viologen molecules to the reduced state (red.) through a redox process, resulting in selective quenching of luminescence owing to spectral resonance between nanocrystals and molecules. A reverse electric field oxidises the reduced viologen molecules, leading to a recovery of upconversion emission. **b** Simplified energy level scheme of lanthanide-activated UCNPs and schematic energy level diagram of viologen molecules in oxidised (Black) and reduced (Blue) forms, illustrating electrically controlled energy transfer from UCNPs to dye molecules. Solid and dashed lines represent electronic transitions.

In this work, inspired by electrochromic organic molecules for optoelectronic applications, such as rewritable, optical labels, and colour-changing metasurfaces^26,27^, we reasoned that fast, multicolour switching of upconversion luminescence should be possible by electrically controlling energy transfer from UCNPs to electrochemically responsive organic molecules. Viologen molecules are the choice of materials for optical switching owing to their good photostability, fast temporal responses, high absorption coefficients, and tailorable absorption wavelengths, as well as ease of structural modification^28–30^. Dynamic modulation of upconversion emission colour and intensity can be achieved with molecular-assisted surface electrochemical tuning (MASET) (Fig. 1a). When pumped with a 980 nm laser, UCNPs exhibit steady luminescence. Upon stimulation with an external electric field, viologen molecules receive electrons and convert from an oxidised state to a reduced state via a redox electrochemical process, resulting in electrically controlled energy transfer from UCNPs to viologen molecules owing to spectral overlap between nanocrystal emission and dye absorption. A recovery of upconversion emission can be rapidly achieved through a reversible redox reaction under a reverse electric field (Fig. 1b).

## Results

### Electrically switchable upconversion luminescence

**Fig. 2:**
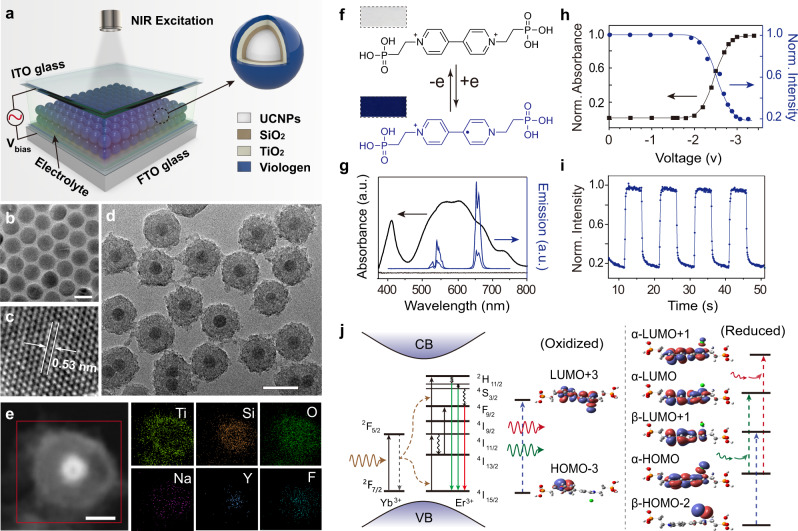
Experimental demonstration of electrically switchable upconversion luminescence. **a** Schematic of an electrochemical cell based on viologen molecule-modified UCNPs. **b**, **c** Representative TEM images of NaYF_4_:Yb/Er nanoparticles under study. Scale bar: 20 nm. **d**, **e** TEM images of NaYF_4_:Yb/Er@SiO_2_@TiO_2_ nanoparticles and corresponding EDX elemental mapping of a single nanoparticle. Scale bar: 50 nm for **d** and 20 nm for **e**. **f** Chemical structures of 1,1’-bis(2-phosphonoethyl)-4,4’-bipyridinium dichloride (PV) in oxidised and reduced forms. Insets show optical images of the oxidised and reduced PV molecule, respectively. **g** Absorption spectra of PV molecules of a hybrid film in oxidised and reduced states (black) and emission spectra of the hybrid film in “bright” and “dark” states (blue). **h** Normalised absorption intensity of PV molecules of a hybrid film at 654 nm (black) and normalised upconversion emission intensity at 654 nm (blue) as a function of the applied potential. **i** Reversible luminescence on/off cycles, recorded at 654 nm under alternating applied potential (±3 V). **j** Simplified energy level diagram of NaYF_4_:Yb/Er nanoparticles and the proposed mechanism underlying the opto-electrochemical modulation.

We experimentally validated the feasibility of the MASET strategy to achieve electrically switchable luminescence and to investigate the involvement of energy transfer between UCNPs and viologen molecules under electrical gating (Fig. 2a). UCNPs with multicolour emission properties were prepared by doping different lanthanide ions (e.g., Yb^3+^, Er^3+^, Tm^3+^, Eu^3+^, and Tb^3+^) into specific layers of hexagonal-phase NaYF_4_ and NaGdF_4_ host materials^31–34^ (Fig. 2b, c and Supplementary Figs. 1, 2). Owing to the nonconductive properties of the host materials, a layer of TiO_2_ was coated onto silica-modified UCNPs to yield semiconducting surfaces (Fig. 2d, e and Supplementary Figs. 3–5). In addition, Ti^4+^ ions possess a strong affinity for highly charged electrochromic molecules^35^. We designed and fabricated a sandwich-like electrochemical cell comprising viologen molecule-modified UCNPs (Fig. 2a). A paste containing TiO_2_-coated NaYF_4_:Yb/Er nanoparticles was deposited on a fluorine-doped tin oxide-coated glass and then calcined to form a semiconducting hybrid film. Under 980 nm laser excitation, the nanoparticle thin film exhibited green and red emission bands at 540 and 654 nm (Supplementary Figs. 6–8). By virtue of its excellent optical properties, an organic molecule, 1,1’-bis(2-phosphonoethyl)-4,4’-bipyridinium dichloride (PV), was selected and synthesised as the electrically active molecule^36^ (Supplementary Figs. 9, 10).

Under appropriate electric field manipulation, PV molecules displayed reversible transformation between their di-cation oxidised form (PV^2+^) and cation-radical reduced form (PV^·+^) (Fig. 2f). Notably, in contrast to the oxidised state, which is transparent to visible and NIR radiation, the reduced state displays a broad absorption band, overlapping considerably with emission bands of NaYF_4_:Yb/Er nanoparticles (Fig. 2g and Supplementary Fig. 11). Under 980 nm irradiation, the hybrid device gave rise to dominant yellow emission peaks at 540 and 654 nm, corresponding to ^2^H_11/2_ → ^4^I_15/2_ and ^4^S_3/2_ → ^4^I_15/2_ transitions of Er^3+^ ions. However, yellow emission was significantly suppressed upon applying an electric field of −3 V. By monitoring voltage-dependent absorbance of PV molecules at 654 nm, we found that electrochemical conversions of PV molecules can be initiated by applying an electric field of −2V. The absorption intensity of PV molecules reached its maximum as the applied voltage was set at −3 V (Fig. 2h and Supplementary Fig. 12). As a result, photoquenching of UCNPs was precisely controlled under an electric field, by which emission intensity gradually decreased with increasing absorbance of PV molecules (Fig. 2h).

The UCNP/PV hybrid system exhibited good durability and fast switching speed with a rise time of 400 ms and a decay time of 300 ms, which are orders of magnitude faster than those of photochromic molecule-mediated upconversion tuning^18,37^. Moreover, no noticeable degradation was observed after 47 writing/erasing cycles, and the “on” and “off” states for each cycle remained the same (Fig. 2i and Supplementary Fig. 12), indicating reversible and stable electric-switching characteristics with excellent fatigue resistance. On the other hand, lifetime decay curves measured for NaYF_4_:Yb/Er nanoparticles in electrochemical cells with or without the electric field remained unchanged (Supplementary Fig. 13), revealing that energy transfer from UCNPs to PV molecules is dominated by a radiative reabsorption process instead of nonradiative Förster resonance energy transfer^38^. Our theoretical investigations suggest that the maximum absorption of oxidised PV molecules is assigned to the electronic transition of HOMO-3→HOMO+3 at 272 nm. In comparison, reduced PV molecules exhibit three dominant electronic transitions of β-HOMO-2 → α-HOMO+1 at 387 nm, α-HOMO → α-HOMO at 521 nm and α-HOMO-2 → α-HOMO+1 at 645 nm (Fig. 2j and Table S1), in good agreement with experimental results.

### Upconversion multicolour editing

**Fig. 3:**
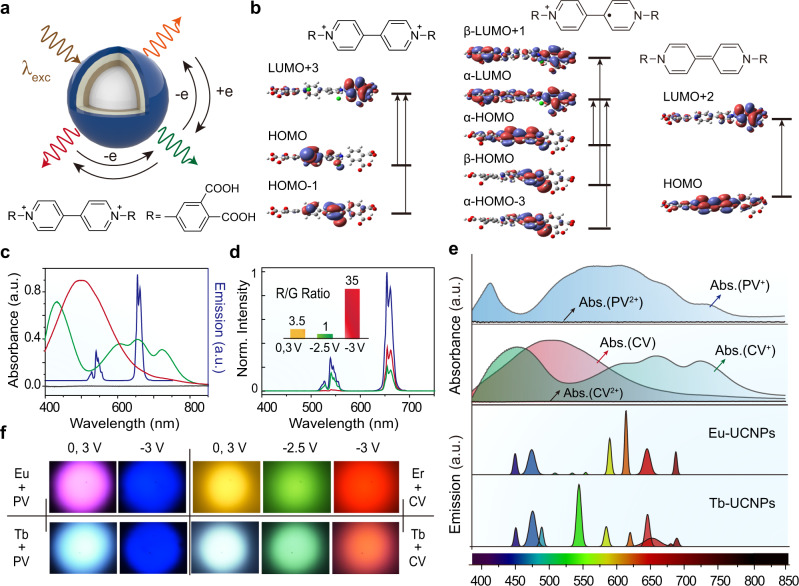
Multicolour luminescence switching of UCNPs through opto-electrochemical modulation. **a** Schematic showing achievement of electrically multicolour switching using UCNPs modified with 1,1’-bis(3,4-dicarboxyphenyl)-4,4’-bipyridinium dichloride (CV) molecules. **b** DFT-calculated molecular orbital diagrams and dominant absorption transitions of 1,1’-bis(3,4-dicarboxyphenyl)-4,4’-bipyridinium dichloride molecule in three reduced and oxidised states. **c** Emission spectrum (blue) of the hybrid film and absorption spectra of CV molecules in the oxidised state (black), first reduced state (green), and second reduced state (red). **d** Emission spectra of the hybrid film under different potentials. Inset shows the red/green intensity ratios obtained under different potentials. **e** Absorption spectra of as-designed viologen molecules (PV and CV) in several reduced and oxidised states and deconvolution of emission spectra of two typical UCNPs (NaGdF_4_:Yb/Tm@NaYF_4_:Eu and NaGdF_4_:Yb/Tm@NaYF_4_:Tb) into individual Gaussian peaks. **f** Upconversion multicolour switching of a wide variety of UCNP/Viologen systems under different potentials.

To further demonstrate the versatility of the MASET strategy to realise multicolour switching (Fig. 3a), we designed a molecule, 1,1’-bis(3,4-dicarboxybenzyl)-4,4’-bipyridinium dichloride (CV), using density functional theory simulations (Table S1 and Supplementary Figs. 14–19). Theoretical predictions and experimental investigations revealed that this molecule features three distinct redox states (CV^2+^, CV^+·^, and CV^··^) under different applied voltages, yielding diverse tunable absorption profiles (Fig. 3b and Supplementary Figs. 20, 21). Electrochemical cells based on CV molecules and NaYF_4_:Yb/Er nanoparticles displayed dominant yellow emission peaks at 540 and 654 nm upon 980 nm excitation, owing to negligible absorption of visible and NIR radiation by the CV molecule in the oxidised state (CV^2+^). However, applying an electric field of −2.5 V markedly suppressed the red emission, but with a slight effect on the green emission at 540 nm. This can be attributed to spectral overlap between the red emission of NaYF_4_:Yb/Er nanoparticles and the absorption band of CV molecules in the first reduced state (CV^+·^) at ~660 nm. Further increasing the voltage to −3 V leads to the conversion of CV molecules from the first (CV^+·^) to the second (CV^··^) reduced state, which features absorption in the blue−green spectral range, but negligible absorption in the red region. As a result, the hybrid device showed red emission (Fig. 3c and Supplementary Fig. 22). These results suggest that by adjusting the voltage within the range of −3 to 3 V, dynamic multicolour editing from yellow to green to red emission is possible using CV molecule-modified NaYF_4_:Yb/Er nanoparticles through modulation of the ratio of red/green emission under different voltages. For example, a low voltage of −2.5 V results in a red/green ratio of 1, giving rise to green emission. Increasing the voltage to −3 V yields intense red emission with a red/green ratio of 35 (Fig. 3d). Based on the MASET strategy, a wide range of colours can be realised at the single-particle level by coupling upconversion nanocrystals possessing tunable emission bands (e.g., NaGdF_4_:Yb/Tm@NaYF_4_:Eu and NaGdF_4_:Yb/Tm@NaYF_4_:Tb) with viologen molecules having variable absorption profiles (Fig. 3e, f and Supplementary Figs. 23–25).

### Nonlinear logic switching circuits

**Fig. 4:**
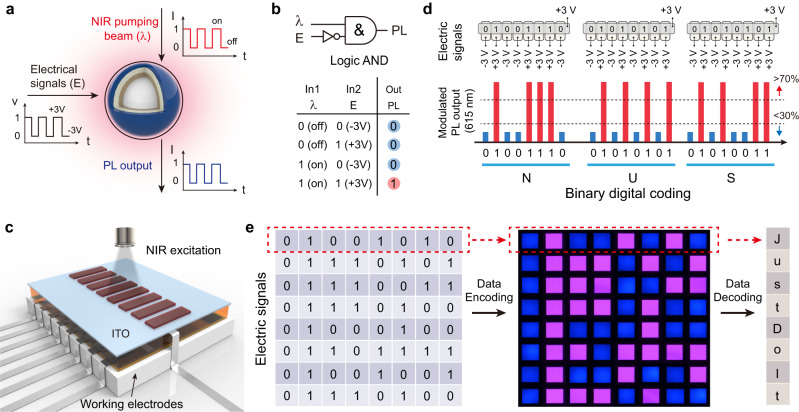
Demonstration of opto-electrochemical logic AND gate using PV-modified UCNPs. **a**, **b** Basic operating principle of logic AND gate based on the Eu-UCNP/PV platform. The nonlinear output signal is programmed by a NIR laser and an alternating electric field. Under NIR illumination, the photonic readout is controlled by alternating the electric field, which initiates the redox reaction of PV molecules. **c** Schematic of an eight-pixeled, UCNP/PV logic gate array, in which each pixel is controlled individually by applying voltages of ±3.0 V. **d** Information processing of electrical inputs carrying the word “NUS” into optical signals using upconversion logic gating. The measured emission intensity of the Eu-UCNP/PV thin film at 615 nm below the 30% or above the 70% threshold represents binary codes, “0” or “1”, respectively. **e** Multiplexed data processing through opto-electrochemical modulation. A string of electrical signals carrying encrypted information of “Just Do It” is converted into visible patterns for signal output.

The ability to electrically modulate anti-Stokes luminescence enables the development of multifunctional nonlinear optoelectronic devices. As a proof-of-concept, electrochemical cells based on PV molecules and NaGdF_4_:Yb/Tm@NaYF_4_:Eu nanoparticles (Eu-UCNPs) were fabricated as opto-electrochemical logic gates for signal processing. In our design, the 980 nm pumping beam (on or off) is considered the optical input (In1). Upon 980 nm excitation, Eu-UCNPs exhibit dominant blue and red emission peaks at 454 and 615 nm, respectively. An alternating electric field (positive or negative) serves as the second input (In2), which can induce reversible spectral resonance between Eu-UCNPs and PV molecules. As such, an upconversion logic AND gate can be constructed with red emission as the output signal. Photonic readout signals, on (1) and off (0), are programmable by modulating NIR excitation and alternating electrical field (Fig. 4a, b).

We next demonstrated this principle by transmitting Roman letters using binary codes of standard eight-bit ASCII characters to digitally encoded optical outputs. Each letter is represented with an eight-digit binary code comprising different combinations of zeros and ones. For instance, the word “NUS” with three capital letters in a specific sequence is composed of data strings of 01001110, 01010101, and 01010011 in binary code. Figure 4c shows a schematic of the Eu-UCNPs/PV based logic gate array with eight pixels representing binary codes of standard eight-bit ASCII characters. Working electrodes (with UCNPs) and counter electrodes (ITO glass) are arranged orthogonally. Cross-sectional components are considered pixels of the matrix, which are controlled individually and independently. In our experimental setting, writing and erasing were performed by applying voltages of −3.0 and +3.0 V. When electrical signals carrying the word “NUS” were transmitted to the eight-pixeled UCNP/PV platform, the device converted electrical signals into upconverted emitting photons, encoding optical outputs of “NUS” in binary digits. Binary codes of “0” or “1” can be expressed by monitoring red emission intensity at 615 nm upon applying voltages of −3.0 and +3.0 V, respectively (Fig. 4d). Moreover, binary digital codes, “0” and “1”, can also be represented with blue and red colours by applying alternating electrical pulses. We further demonstrated conversion of electrical signals “Just Do It” to high-fidelity photonic output through upconversion logic AND gate (Fig. 4e). Compared with DNA- and quantum dot-based switching circuits with readout based on changes in structural configuration or monochromatic emission intensity^39,40^, the multicolour switching characteristic of our system has advantages such as direct visualisation and easy identification of encrypted information.

## Discussion

In summary, we have experimentally demonstrated precise control over anti-Stokes luminescence, based on coupling of lanthanide-activated nanoparticles and electrochemically responsive viologen molecules. By tailoring orbital energy levels of molecules under electric field modulation, the absorption profile of viologen molecules can be adjusted on-demand, enabling electrically tunable spectral resonance between UCNPs and dye species and ultimately resulting in dynamic multicolour editing of anti-Stokes luminescence. This molecularly engineered UCNP platform also enables opto-electrochemical signal processing for rapid, high-fidelity, and far-field communication. These results not only allow active control over anti-Stokes luminescence under low electric fields and pumping thresholds, but will also benefit the future development of nonlinear photonic devices for applications in quantum computing, multiplexed sensing, data encryption, and potentially many others.

## Methods

### Nanocrytal synthesis

Upconversion nanoparticles were synthesised using a co-precipitation method. Upconversion microrods were synthesised by a hydrothermal method. Detailed experimental procedures for the preparation of different types of nanoparticles/microrods are provided in the Supplementary Methods.

### Preparation of ligand-free NaLnF_4_ nanocrystals

As-synthesised oleic acid-capped upconversion nanoparticles/microrods were dispersed in the mixture containing 1 mL of ethanol and 1 mL of 2-M HCl solution. The resulting mixture was ultrasonicated for 10 min to remove oleic acid ligands. Ligand-free nanoparticles were collected by centrifugation, washed with ethanol/deionized water several times, and re-dispersed in deionized water.

### Preparation of NaLnF_4_@SiO_2_@TiO_2_ nanoparticles

To a 50-mL round-bottom flask containing 15 mL of ethanol and 5 mL of deionized water were added 250-mg PVP under stirring. After dissolving the PVP surfactant, ligand-free nanoparticles (0.2f poly(vinyl pyrrolidone) (PVP) mmol) were added. After stirring for 30 min, a 300-μL ethanol solution containing 50-μL tetraethyl orthosilicate was added. The resulting mixture was reacted overnight under stirring. NaLnF_4_@SiO_2_ nanoparticles were collected via centrifugation at 16,500 rpm for 30 min, followed by washing with ethanol twice. To a 50-mL round-bottom flask containing 15 mL of methanol and 5 mL of deionized water were added 250-mg PVP under stirring. After dissolving the PVP surfactant, a solution of as-prepared silica-modified NaLnF_4_ core-shell nanoparticles was added. After stirring for 30 min, a 200-μL methanol solution containing titanium diisopropoxide bis(acetylacetonate) precursor was added. The resulting mixture was kept overnight under stirring. NaLnF_4_@SiO_2_@TiO_2_ nanoparticles were collected by centrifugation and washed with ethanol and deionized water several times.

### Preparation of NaLnF_4_@TiO_2_ microrods

Typically, a mixture solution containing 2 mL of PVP-40 (0.2 g/mL) aqueous solution and 5 mL of ethanol was added 0.1 mmol as-prepared ligand-free NaLnF_4_ microrods under stirring. A 2 mL fresh-prepared TiF_4_ (0.04 M) aqueous solution was dropwise added into the above solution. The mixture solution was then transferred into a Teflon-lined autoclave and heated to 180 °C for 6 h. The resulting products were collected by centrifugation and washed with ethanol and deionized water several times.

### Synthesis of 1,1’-bis(2-phosphonoethyl)-4,4’-bipyridinium dichloride

To a 100-mL round-bottom flask containing 50 mL of water were added 4,4’-bipyridine (2.35 g, 15 mmol) and diethyl-2-bromoethyl phosphonate (7.5 g, 30 mmol). Upon refluxing for 48 h, 8 mL of concentrated hydrochloric acid was added to the reaction mixture and refluxed for another 24 h. After cooling to room temperature, the reaction mixture was concentrated to 5 mL, followed by dropwise addition of 2-propanol. Upon stirring in an ice bath, the resulting products were filtered, washed with cold 2-propanol three times, and dried in a vacuum oven. ^1^H NMR (300 MHz, D_2_O) δ 9.08 (d, *J* = 6.3 Hz, 4H), 8.46 (d, *J* = 6.4 Hz, 4H), 4.85 (dt, *J* = 12.4, 7.8 Hz, 4H), 2.38 (dt, *J* = 17.5, 7.7 Hz, 4H).

### Synthesis of 1,1’-bis(3,4-dicarboxybenzyl)-4,4’-bipyridinium dichloride

To a 50-mL flask charged with acetonitrile were added 4,4’-bipyridine (2.5 g, 16 mmol) and 2,4-dinitro-chlorobenzene (7.0 g, 34 mmol). The reaction mixture was refluxed at 80 °C for 12 h under stirring. After reaction, the resulting 1,1’-bis(2,4-dinitrophenyl)-bipyridinium dichloride was filtered and washed with acetonitrile three times and with diethyl ether four times, and dried in a vacuum oven. Compound 4-aminophthalic acid in ethanol and a stoichiometric amount of 1,1’-bis(2,4-dinitrophenyl)-bipyridinium dichloride were added into the flask. The mixture was refluxed for 24 h under stirring. The solution was evaporated after filtration and extraction. The residue was dissolved in methanol and added to diethyl ether. Precipitated products were collected by filtration, washed with ethyl acetate/diethyl ether several times, and dried in a vacuum oven. ^1^H NMR (500 MHz, DMSO) δ 9.79 (d, *J* = 7.0 Hz, 4H), 9.16 (d, *J* = 7.1 Hz, 4H), 8.37 (d, *J* = 2.3 Hz, 2H), 8.21 (dd, *J* = 8.3, 2.4 Hz, 2H), 8.09 (d, *J* = 8.3 Hz, 2H). ^13^C NMR (126 MHz, DMSO) δ 168.21, 167.33, 149.64, 146.79, 143.50, 136.58, 134.46, 130.12, 128.13, 127.06, 126.25.

### Fabrication of opto-electrochemical devices

In brief, the prepared NaLnF_4_@SiO_2_@TiO_2_ nanoparticles were mixed with a TiO_2_ paste, and the mixture was dispersed in ethanol containing 5 wt.% PEG to form a stock slurry under stirring overnight. The fluorine-doped tin oxide (FTO) (15 Ω cm^−2^) glass was cleaned by oxygen plasma for 20 min. A compact TiO_2_ layer was formed on the FTO glass by spin-coating the mixed solution (0.1 M) of titanium diisopropoxide bis(acetylacetonate) in 1-butanol on the substrate and then annealed at 400 °C for 30 min. The UCNPs@SiO_2_@TiO_2_ hybrid film was prepared on the TiO_2_-coated FTO glass by a doctor blade method. The hybrid film was then dried at room temperature for 30 min and then heated at 80 °C for another 30 min, followed by annealing at 400 °C for 20 min in a vacuum tube furnace. The hybrid film was immersed in a 50-mM aqueous solution of viologen molecules for 12 h. The substrate was washed with ethanol and then dried in a vacuum oven overnight. To construct the electrochemical cell, UCNPs@SiO_2_@TiO_2_ nanoparticle-based working electrode and another ITO counter electrode (6 Ω cm^−2^) were joined using double-sided tape. A solution of 0.5 M LiClO_4_ in propylene carbonate was injected as the electrolyte into the cell after removal of air.

### Physical measurements

Powder X-ray diffraction data were obtained from a Bruker D8 Advance diffractometer using graphite-monochromatized CuKα radiation (*λ* = 1.5406 Å). Transmission electron microscopy images were collected from a JEOL-JEM 2100 F electron microscope. Scanning electron microscopy images were taken on a JEOL-JSM-6701F electron microscope. ^1^H and ^13^C NMR spectra were recorded on a Bruker Advance 500 spectrometer at ambient temperature. UV-vis absorption spectra were recorded with a SHIMADZU ultraviolet-3600 spectrophotometer. Electrochemical characterisation of the viologen molecule-modified electrode employed a CS310 potentiostat with a Ag/AgCl/1 M KCl (aq) reference electrode.

### Optical characterisations

Optical characterisations were carried out using the custom-built microscope capable of luminescence imaging and spectroscopy. For photoluminescence measurements, a 980 nm continuous-wave excitation laser is coupled to an optical microscope and focused onto the samples. The 980 nm excitation laser with the power intensity of ~200 W/cm^2^ was used for all the experiments. The photoluminescence is collected through a microscope objective and passed through an 800 nm short-pass fluorescence filter and sent onto a fibre-coupled Ocean Spectrometer.

## Supplementary information

Supplementary Information

## Data Availability

The authors declare that the data that support the findings of this study are available within the article and its Supplementary Information files. All other relevant data are available from the corresponding author upon reasonable request.
