# Supplementary material for: Dynamic upconversion multicolour editing enabled by molecule-assisted opto-electrochemical modulation
Source: Nat Commun. 2021 Apr 1;12:2022. doi: 10.1038/s41467-021-22387-7 (PMC8016979; doi:10.1038/s41467-021-22387-7)
Supplement: Supplementary file 1 — Supplementary Information [file 41467_2021_22387_MOESM1_ESM.pdf]

Materials and methods.

Materials.

$Y(CH_3CO_2)_3{\bullet}H_2O$ (99.9%), $Yb(CH_3CO_2)_3{\bullet}H_2O$ (99.9%), $Er(CH_3CO_2)_3{\bullet}H_2O$ (99.9%), $Gd(CH_3CO_2)_3{\bullet}H_2O$, (99.9%), $Tm(CH_3CO_2)_3{\bullet}H_2O$ (99.9%), $Tb(CH_3CO_2)_3{\bullet}H_2O$ (99.9%), $Eu(CH_3CO_2)_3{\bullet}H_2O$ (99.9%), oleic acid (90%), 1-octadecene (90%), sodium hydroxide (NaOH; >98%), ammonium fluoride (NH_4F; >98%), poly(vinyl pyrrolidone) (PVP; MW ≈ 55,000), poly(vinyl pyrrolidone) (PVP; MW ≈ 40, 000), poly(ethylene glycol) (PEG; MW ≈ 20,000), titanium diisopropoxide bis(acetylacetonate) (TDAA), titanium fluoride (TiF_4), tetraethyl orthosilicate (TEOS), cyclohexane (anhydrous; >99.5%), Acetonitrile (anhydrous; 99.8%), 1-butanol (99.8%), 2-propanol (anhydrous; 99.5%), diethyl ether (anhydrous; ≥99.7%), ethyl acetate (anhydrous; 99.8%), 4,4′-bipyridine (98%), $LiClO_4$ (99.99%), propylene carbonate (anhydrous; 99.7%), diethyl-2-bromoethyl phosphonate (97%), 2,4-dinitro-chlorobenzene (97%), and 4-aminophthalic acid (97%) were all purchased from Sigma-Aldrich and used as received without further purification.

Nanocrystal Synthesis.

Synthesis of $NaYF_4$:Yb/Er (18/2 mol%) nanoparticles. Nanoparticles were synthesized using a co-precipitation method. Typically, to a 50-mL flask containing 3 mL of oleic acid, 1.8 mL of $Y(CH_3CO_2)_3$, 160 µL of $Yb(CH_3CO_2)_3$ and 40 µL of $Er(CH_3CO_2)_3$ were added. The reaction mixture was heated at 150 °C for 30 min under stirring to remove the water content from the solution. Then, 7 mL of 1-octadecene was then quickly introduced into the above mixture, followed by heating at 150 °C for another 30 min before cooling to 50 °C. Shortly after that, 1.6 mmol NH_4F and 1 mmol NaOH methanol solution were introduced to the resulting mixture under stirring for another 30 min. Upon evaporation of methanol, the solution was heated to 290 °C under an argon atmosphere for 1.5 h and then cooled to room temperature. The resulting nanoparticles were precipitated by addition of ethanol, collected by centrifugation, washed with ethanol twice, and re-dispersed in 4 mL of cyclohexane.

Synthesis of $NaGdF_4$:Yb/Tm (49/1 mol%) core nanoparticles. The synthesis procedure of $NaGdF_4$:Yb/Tm (49/1 mol%) nanoparticles is similar to that presented above, except that 4 mL of oleic acid, 6 mL of 1-octadecene, 1 mL of $Gd(CH_3CO_2)_3$, 0.98 mL of $Yb(CH_3CO_2)_3$ and 20 μL of $Tm(CH_3CO_2)_3$ were used. The resulting nanoparticles were collected by centrifugation, washed with ethanol twice, and re-dispersed in 4 mL of cyclohexane.

Synthesis of $NaGdF_4$:Yb/Tm(49/1 mol%)@$NaYF_4$:Eu (15%) nanoparticles. The core@shell nanoparticles were prepared by the co-precipitation method through the use of the pre-synthesized core nanoparticles as seeds. The coating procedure of $NaYF_4$:Eu (15%) shell is similar to that of core nanoparticles described above, except that aqueous solutions of 1.7 mL of $Y(CH_3CO_2)_3$ and 300 μL of $Eu(CH_3CO_2)_3$ together with core nanoparticles dispersed in cyclohexane, and a methanol solution of NH_4F and NaOH were used to initiate an epitaxial shell growth process. The resulting core@shell nanoparticles were collected by centrifugation, washed with ethanol, and re-dispersed in 4 mL of cyclohexane.

Synthesis of $NaGdF_4$:Yb/Tm(49/1 mol%)@$NaYF_4$:Tb (15%) nanoparticles. The synthesis procedure of $NaGdF_4$:Yb/Tm (49/1 mol%) core nanoparticles is presented above. The coating procedure of $NaYF_4$:Tb (15%) shell is similar to that of core nanoparticles described above, except that a mixture containing aqueous solutions of 1.7 mL of $Y(CH_3CO_2)_3$ and 300 μL of $Tb(CH_3CO_2)_3$ and core nanoparticles dispersed in cyclohexane, as well as a methanol solution of NH_4F and NaOH were used to initiate an epitaxial shell growth process. The resulting core@shell nanoparticles were collected by centrifugation, washed with ethanol, and re-dispersed in 4 mL of cyclohexane.

Synthesis of $NaYF_4$:Yb/Er microrods. Microrods were synthesized by a hydrothermal method. A NaOH aqueous solution (1.5 mL, 0.2g/mL) was added to a mixture containing 5 mL ethanol and 5 mL oleic acid under stirring, followed by addition of 2 mL of 0.2-M $RECl_3$ (RE = Y, Yb, Er; 80/18/2) and 1 mL of 2-M NH_4F aqueous solution. The solution was transferred into a Teflon-lined autoclave, heated at 220 °C for 10 h, and then cooled to room temperature. The resulting rods were collected by centrifugation and washed twice with ethanol and deionized water.

Preparation of $NaLnF_4@TiO_2$ nanoparticles. To a 50-mL round bottom flask containing 15 mL of methanol and 5 mL of deionized water were added 250-mg PVP-55 under stirring. After dissolving the PVP surfactant, ligand-free nanoparticles (0.2 mmol) were added. After stirring for 30 min, a 200-μL methanol solution containing titanium diisopropoxide bis(acetylacetonate) precursor was added. The reaction was allowed to proceed for 12 h at room temperature under stirring. The core@shell nanoparticles were collected via centrifugation, followed by washing with ethanol twice and deionized water twice.

Characterization of 1,1'-bis(3,4-dicarboxybenzyl)-4,4'-bipyridinium dichloride and 1,1'-bis(3,4-dimethyl phthalate)-4,4'-bipyridinium dichloride. ^{1}H-NMR and ^{13}C-NMR for the 1,1'-bis(3,4-dicarboxybenzyl)-4,4'-bipyridinium dichloride were showed in Supplementary Figs. S15-S16. However, the HRMS data of 1,1'-bis(3,4-dicarboxybenzyl)-4,4'-bipyridinium dichloride are difficult to be obtained due to the high reactivity of the carboxybenzyl functional groups under high temperature and pressure conditions. To further confirm the dominant chemical structure, the corresponding 1,1'-bis(3,4-dimethyl phthalate)-4,4'-bipyridinium dichloride was synthesized through esterification of 1,1'-bis(3,4-dicarboxybenzyl)-4,4'-bipyridinium dichloride. ^{1}H-NMR, ^{13}C-NMR and high-resolution mass spectrometry for the 1,1'-bis(3,4-dimethyl phthalate)-4,4'-bipyridinium dichloride were showed in Supplementary Figs. S17-S19.

Simulation section. The quantum calculations including geometry optimization and vertical transition were performed using Gaussian 19 program[1] (Rev A.03). The ground-state geometries were fully optimized using B3LYP functional along with 6-311G** basis set. For the calculation of vertical transition energy, a larger basis set, namely def2-TZVP, was employed. GaussView 5 is used to visualize the molecular structures[2].

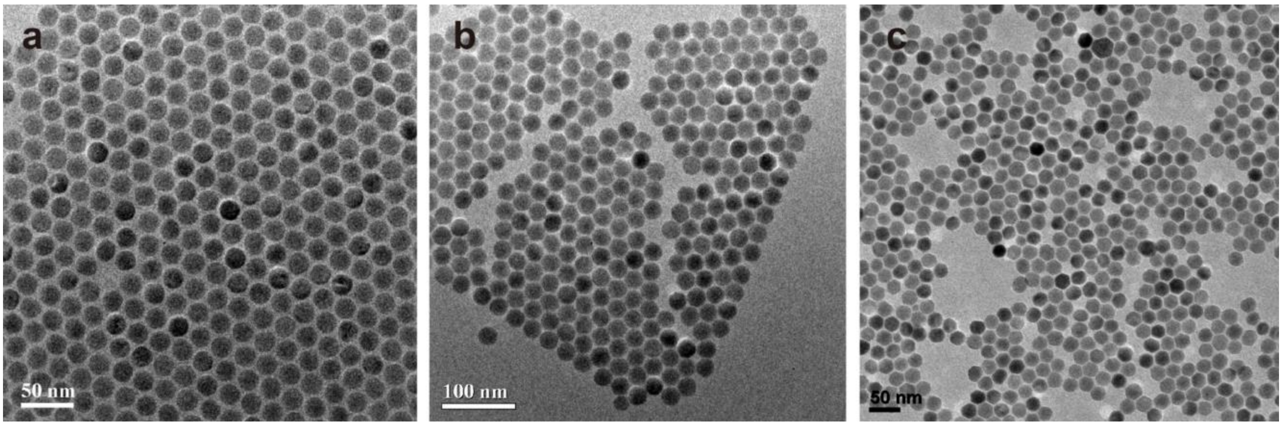

Supplementary Figure 1. TEM images of **a**, $NaYF_4$:Yb/Er (18/2 mol%); **b**, $NaGdF_4$:Yb/Tm (49/1 mol%)@$NaYF_4$:Eu (15 mol%) and **c**, $NaGdF_4$:Yb/Tm (49/1 mol%)@$NaYF_4$:Tb (15 mol%) nanoparticles.

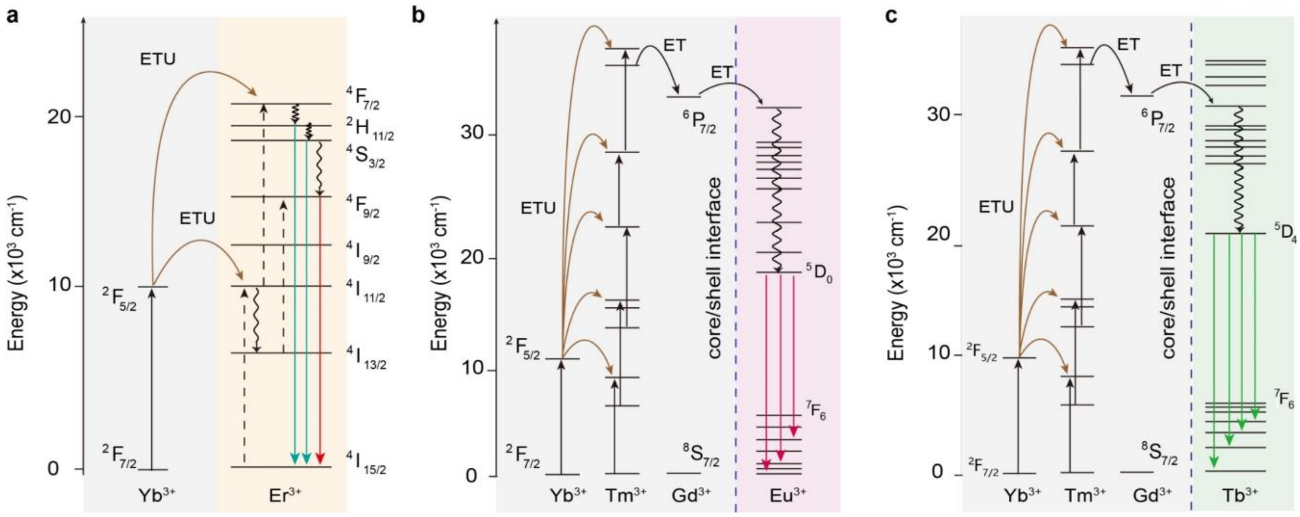

Supplementary Figure 2. Proposed energy transfer mechanisms in **a**, $NaYF_4$:Yb/Er(18/2 mol%); **b**, $NaGdF_4$:Yb/Tm (49/1 mol%)@$NaYF_4$:Eu (15 mol%); and **c**, $NaGdF_4$:Yb/Tm (49/1 mol%)@$NaYF_4$:Tb (15 mol%) nanoparticles.

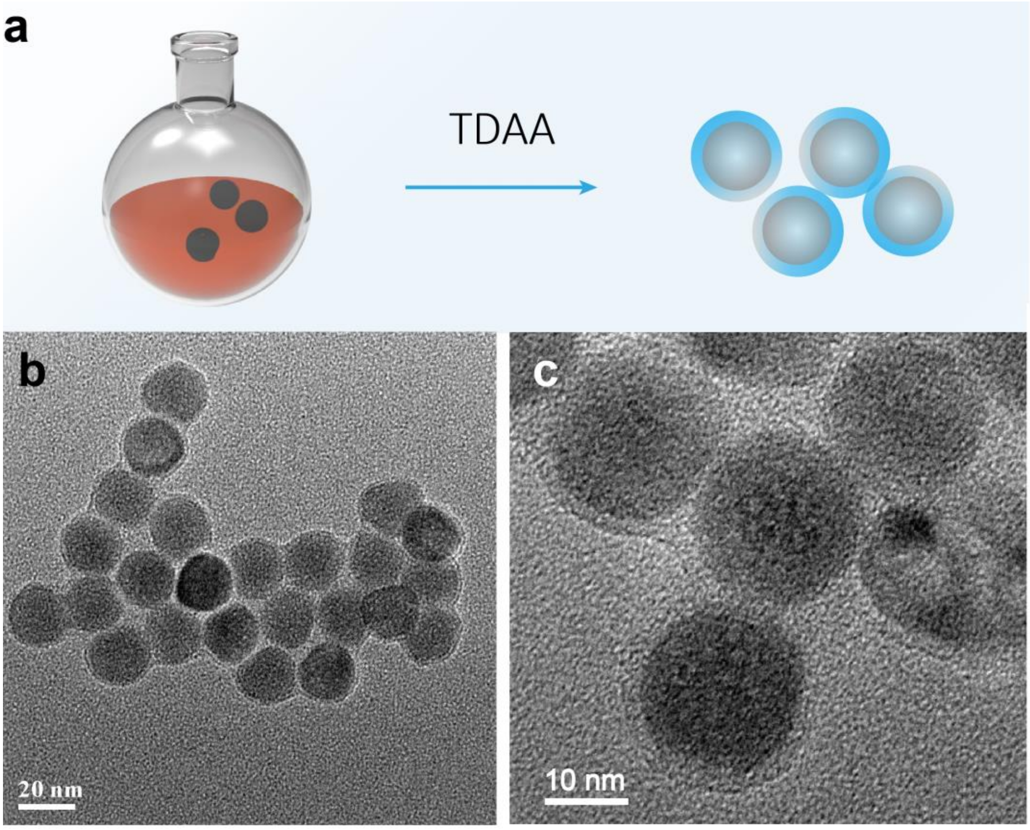

Supplementary Figure 3. a, Schematic illustration of the formation of a thin layer of TiO_2 on UCNPs. **b,c**, TEM images of TiO_2-coated UCNPs.

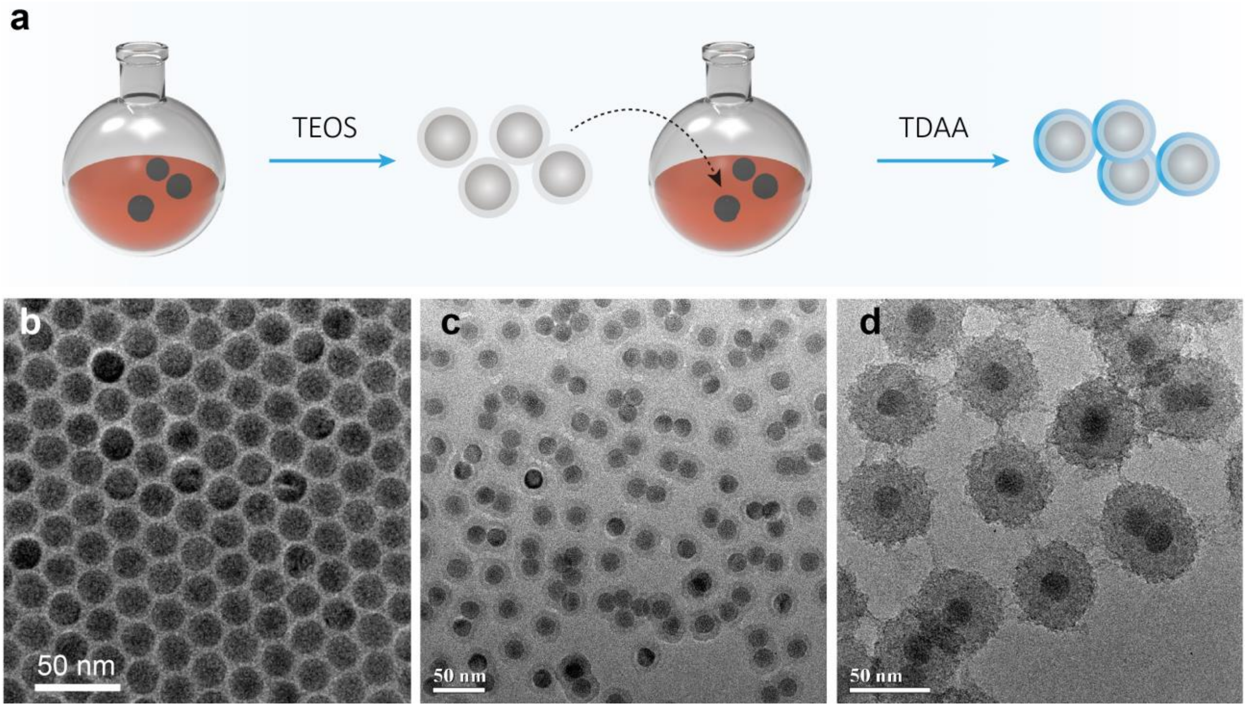

Supplementary Figure 4 a, Schematic illustration of UCNP@SiO_2@TiO_2 nanoparticle synthesis. **b-d**, TEM images of UCNPs, UCNP@SiO_2 and UCNP@SiO_2@TiO_2 nanoparticles, respectively. These results suggest that the introduction of a silica nanoshell facilitates a thick coating of TiO_2 on UCNPs.

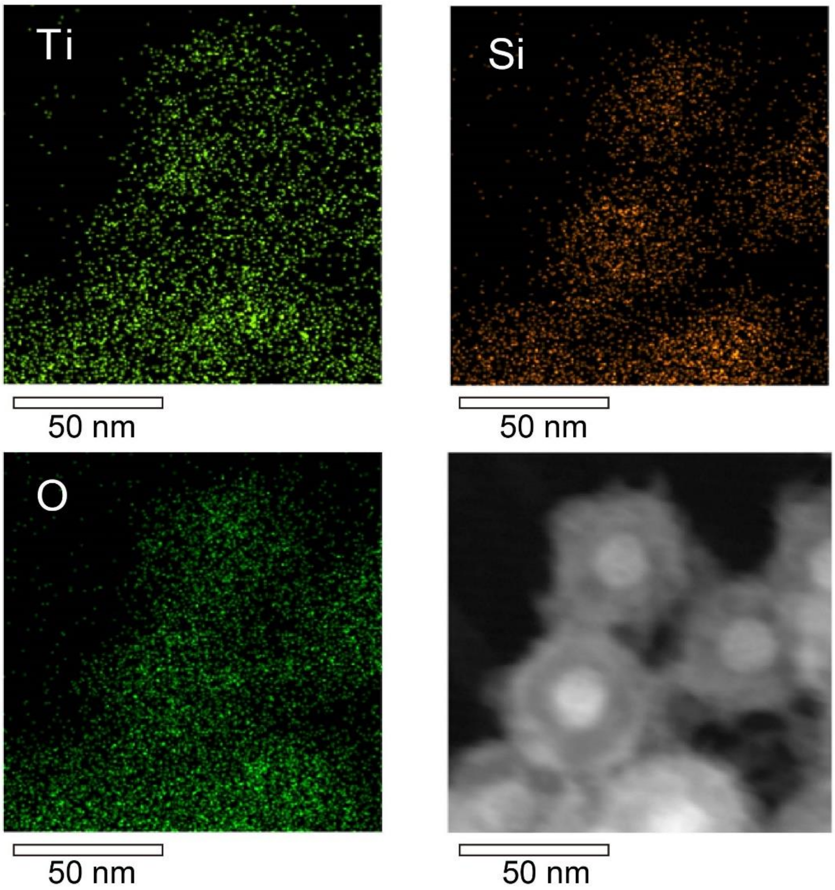

Supplementary Figure 5. TEM image and corresponding EDX elemental mapping of UCNP@SiO_2@TiO_2 nanoparticles.

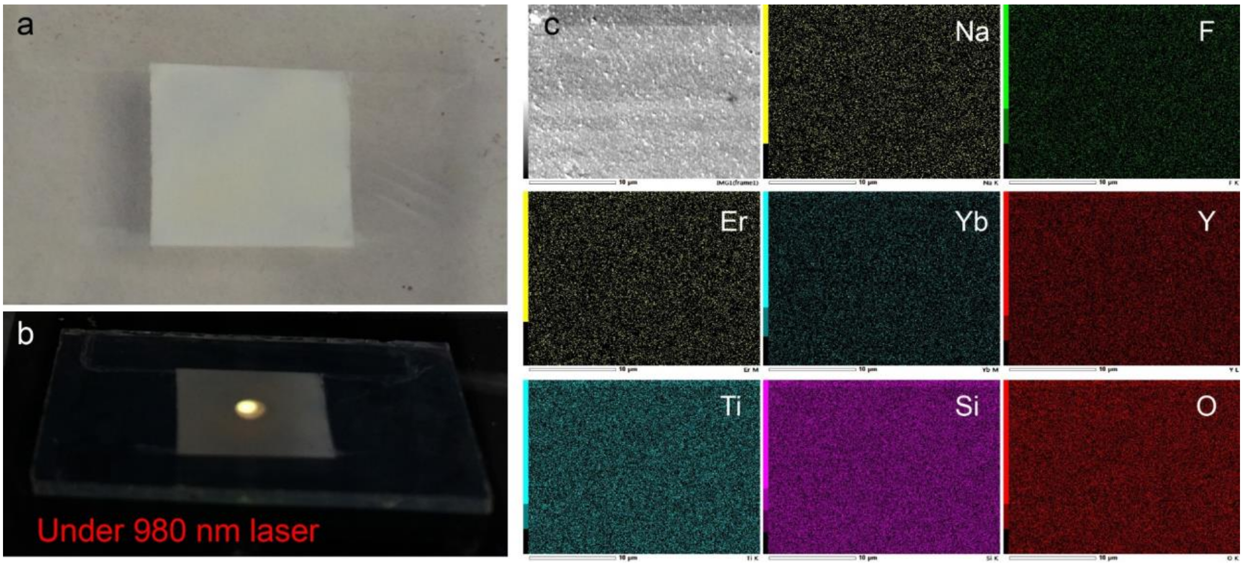

Supplementary Figure 6. a, Optical image of a thin film comprising $NaYF_4$:Yb/Er@SiO_2@TiO_2 nanoparticles. **b,** Photoluminescence imaging of the nanoparticle film under 980-nm illumination. **c**, SEM images and corresponding EDX elemental mappings of the nanoparticle film.

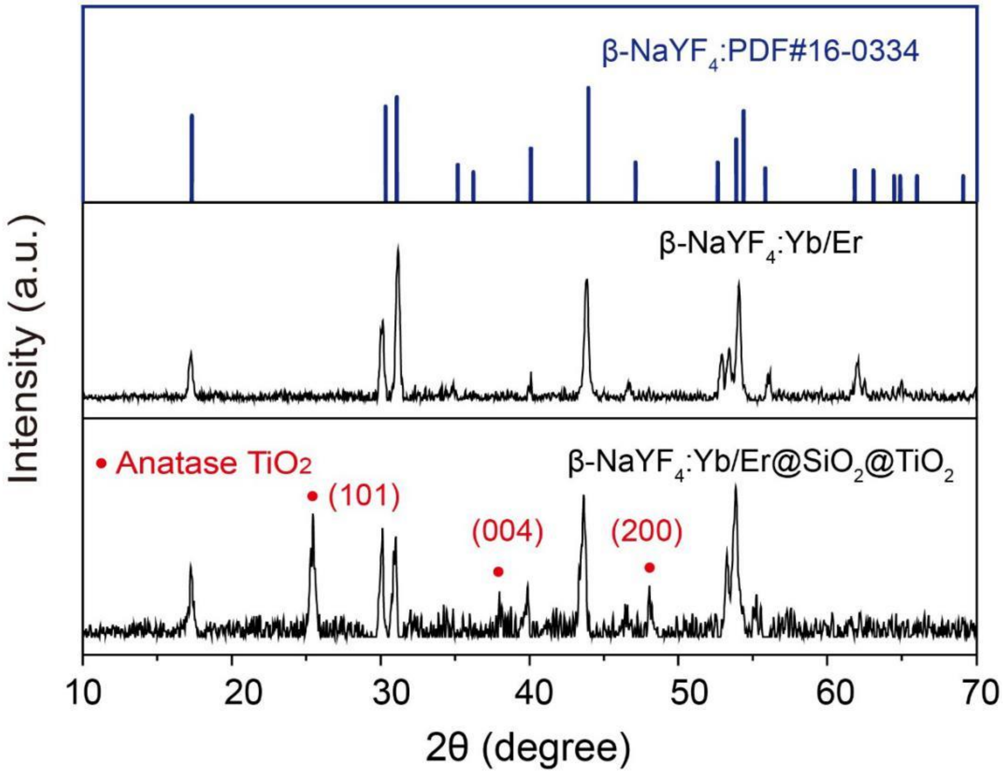

Supplementary Figure 7. XRD patterns of $NaYF_4$:Yb/Er and $NaYF_4$:Yb/Er@SiO_2@TiO_2 nanoparticles, indicating the hexagonal phase of β-$NaYF_4$:Yb/Er core and anatase phase of the TiO_2 shell.

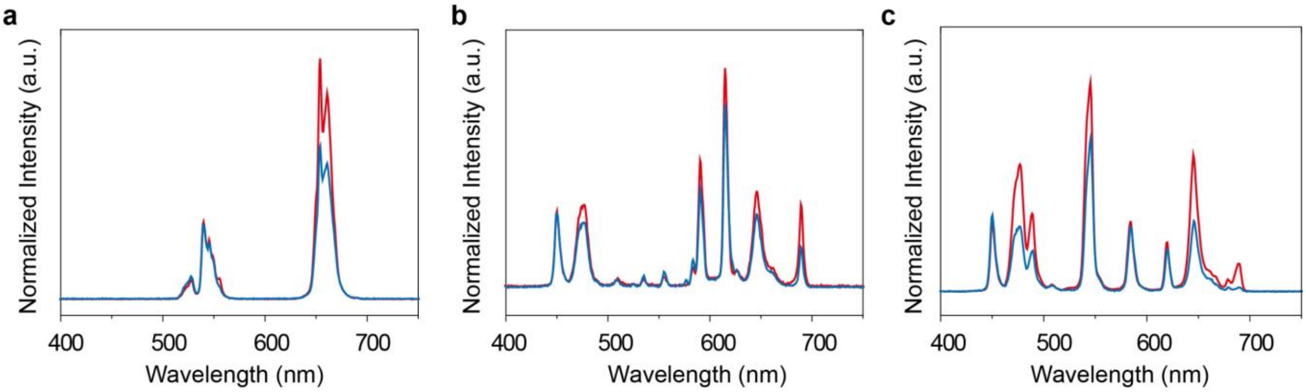

Supplementary Figure 8. Upconversion emission spectra of thin films comprising: **a**, $NaYF_4$:Yb/Er(18/2 mol%); **b**, $NaGdF_4$:Yb/Tm(49/1 mol%)@$NaYF_4$:Eu(15 mol%); and **c**, $NaGdF_4$:Yb/Tm(49/1 mol%)@$NaYF_4$:Tb(15 mol%) nanoparticles, recorded under 980-nm excitation before (blue) and after (red) annealing.

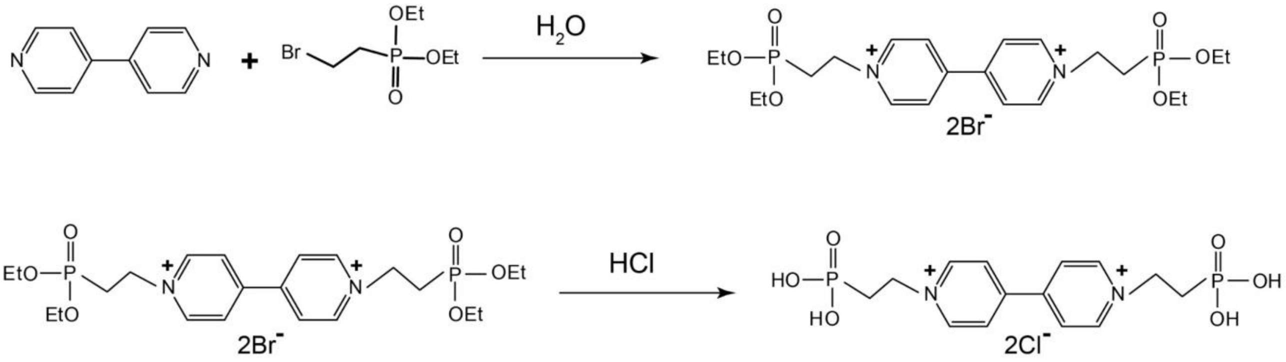

Supplementary Figure 9. Schematic of chemical synthesis procedure for 1,1'-bis(2-phosphonylethyl)-4,4'-bipyridinium dichloride.

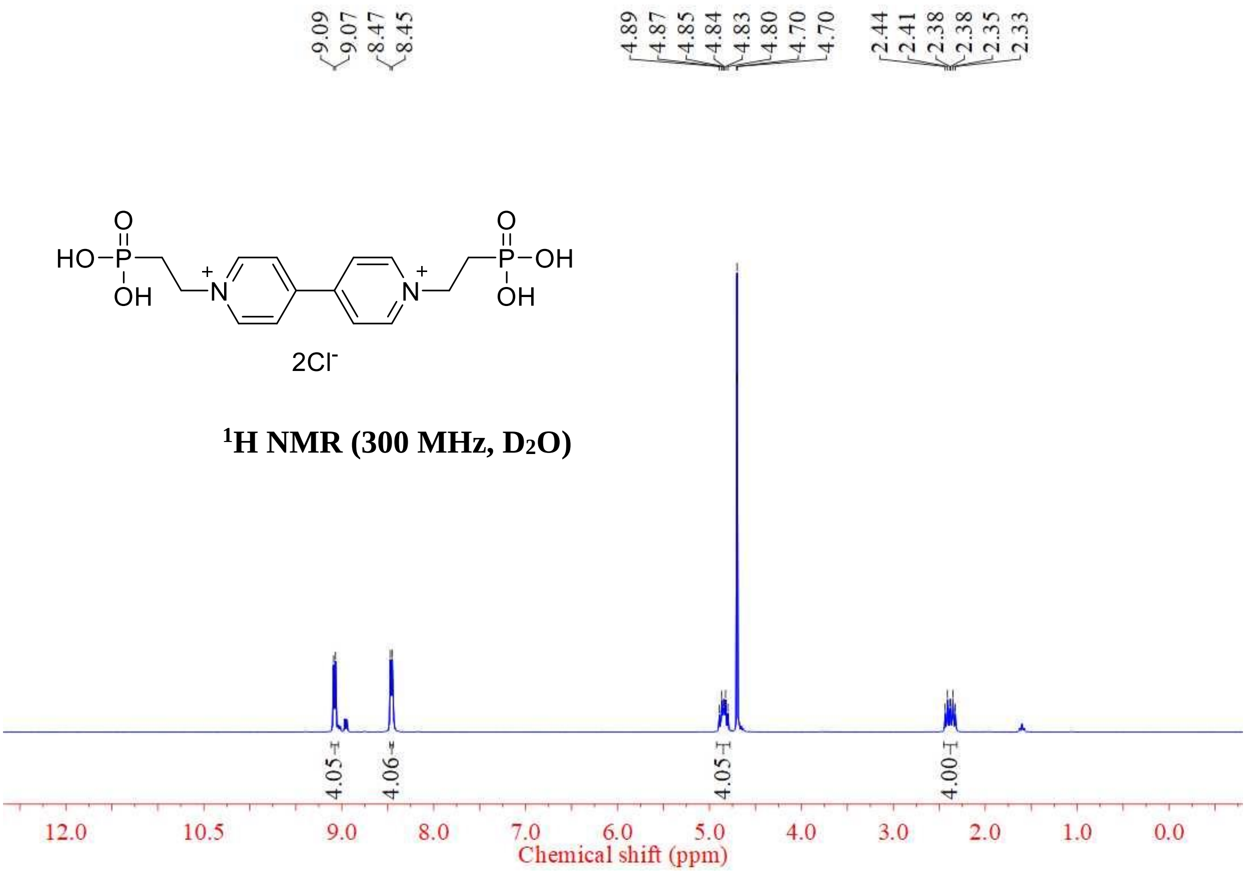

Supplementary Figure 10. ^{1}H-NMR spectrum of 1,1'-bis(2-phosphonylethyl)-4,4'-bipyridinium dichloride. ^{1}H NMR (300 MHz, D_2O) δ 9.08 (d, *J* = 6.3 Hz, 4H), 8.46 (d, *J* = 6.4 Hz, 4H), 4.85 (dt, *J* = 12.4, 7.8 Hz, 4H), 2.38 (dt, *J* = 17.5, 7.7 Hz, 4H).

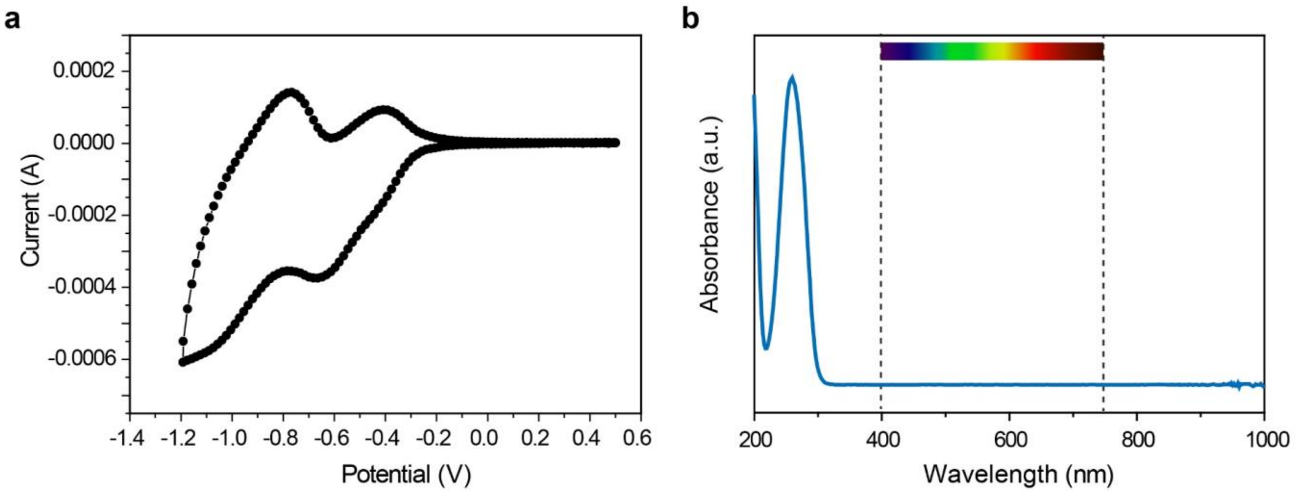

Supplementary Figure 11. **a**, Cyclic voltammogram of 1,1'-bis(2-phosphonylethyl)-4,4′-bipyridinium dichloride-modified, nanostructured TiO_2 electrode versus Ag/AgCl (1.0 M KCl) reference electrode in an electrolyte consisting $LiClO_4$ (0.5 M) in propylene carbonate, indicating the presence of intermediates in redox reactions and the good reversibility of the reaction. **b**, Absorption spectrum of 1,1'-bis(2-phosphonylethyl)-4,4′-bipyridinium dichloride molecule at oxidized state as recorded in an aqueous solution.

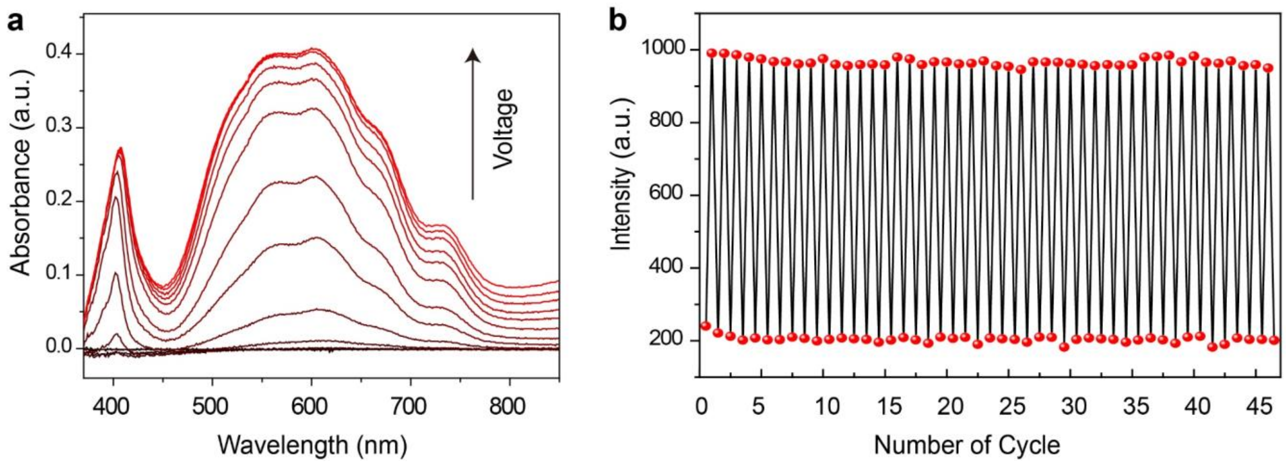

Supplementary Figure 12. **a**, Potential-dependent optical absorption spectra of 1,1’-bis(2-phosphonylethyl)-4,4′-bipyridinium dichloride molecules anchored onto the nanoparticle film. **b**, Reversible photoluminescence on/off cycles recorded at 654 nm by applying alternating potentials of ±3 V.

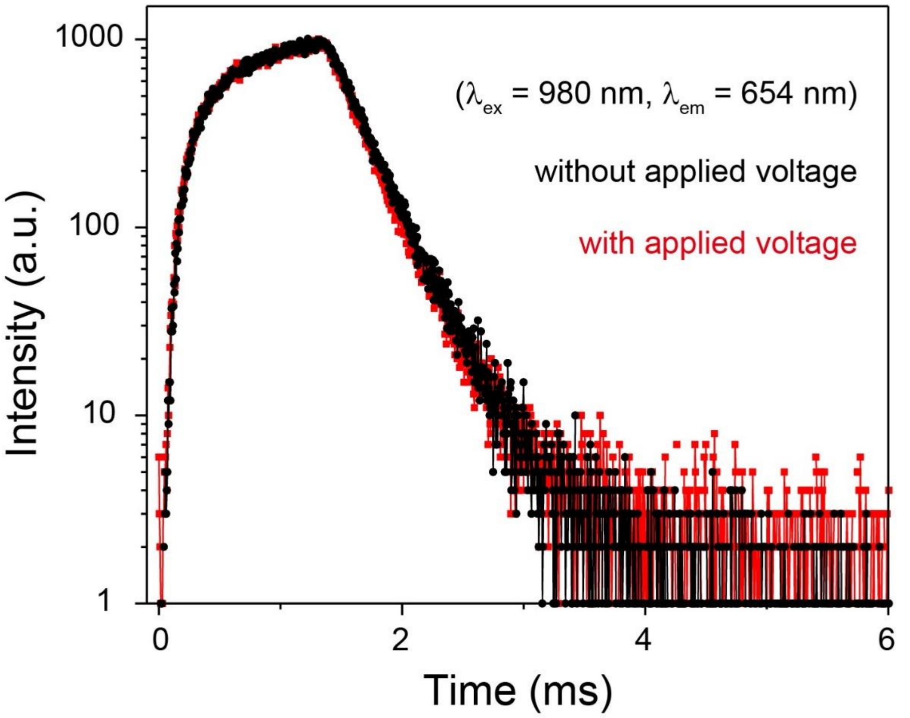

Supplementary Figure 13. Lifetime decay curves measured for $NaYF_4$:Yb/Er nanoparticles in an electrochemical cell without (black) or with (red) an applied potential of -3V (λ_{ex} = 980 nm, λ_{em} = 654 nm).

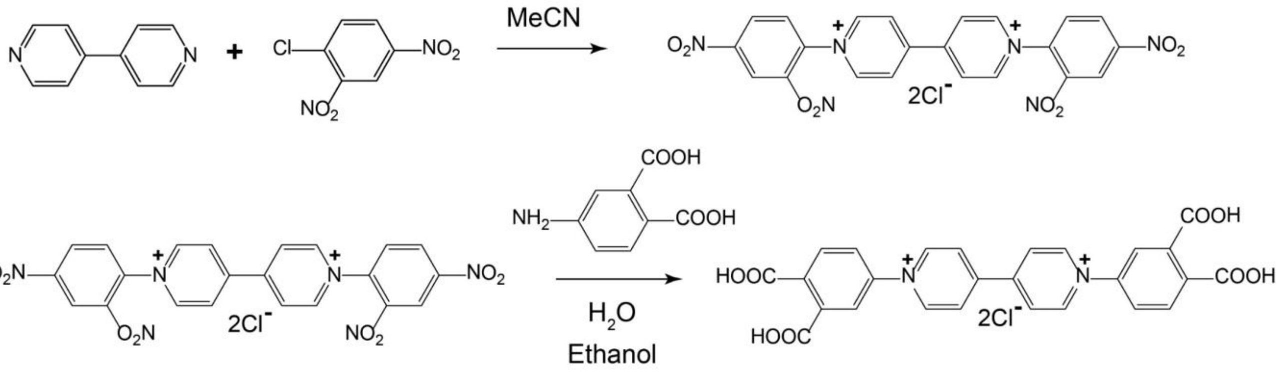

Supplementary Figure 14. Schematic of chemical synthesis procedure for 1,1'-bis(3,4-dicarboxybenzyl)-4,4'-bipyridinium dichloride.

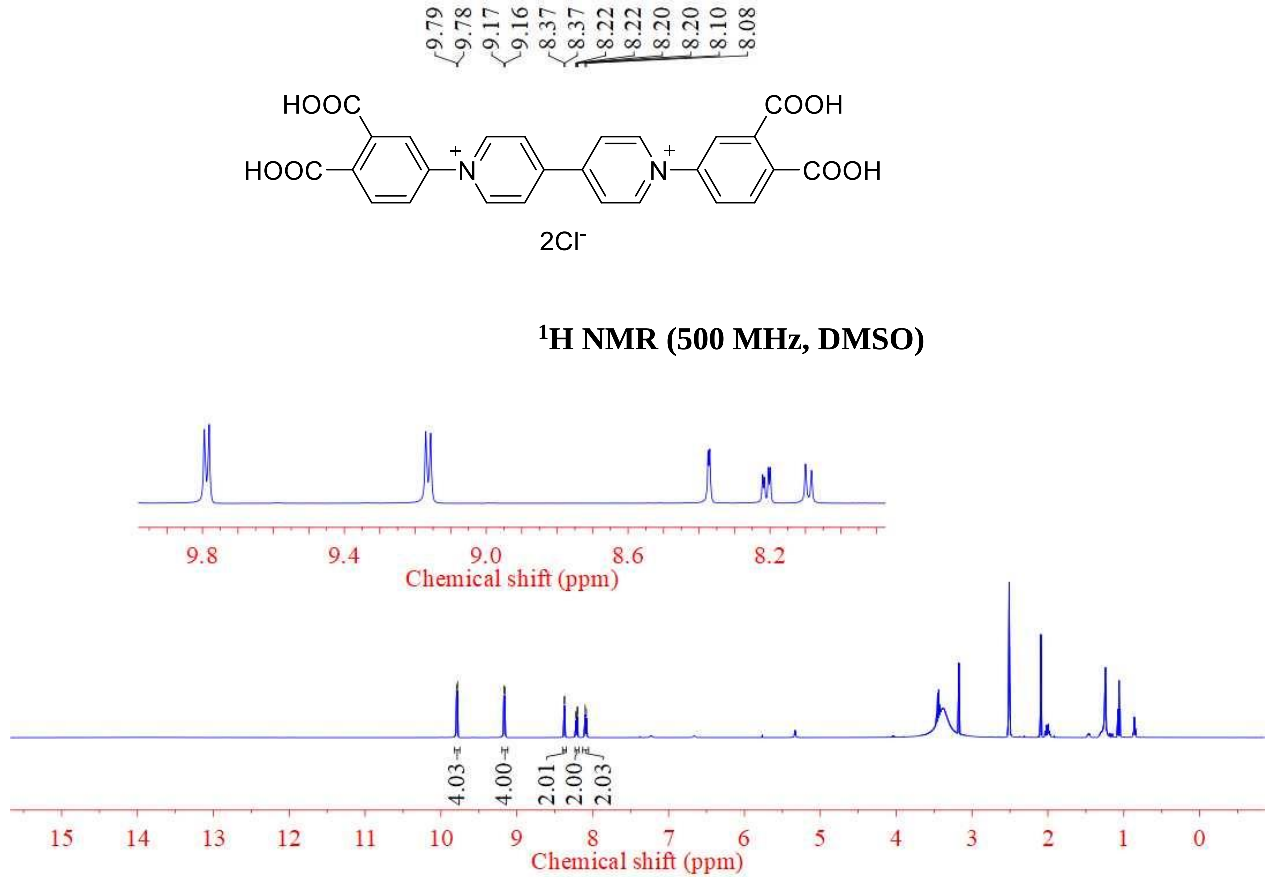

Supplementary Figure 15. ^{1}H-NMR spectrum of 1,1'-bis(3,4-dicarboxybenzyl)-4,4'-bipyridinium dichloride. ^{1}H NMR (500 MHz, DMSO) δ 9.79 (d, J = 7.0 Hz, 4H), 9.16 (d, J = 7.1 Hz, 4H), 8.37 (d, J = 2.3 Hz, 2H), 8.21 (dd, J = 8.3, 2.4 Hz, 2H), 8.09 (d, J = 8.3 Hz, 2H).

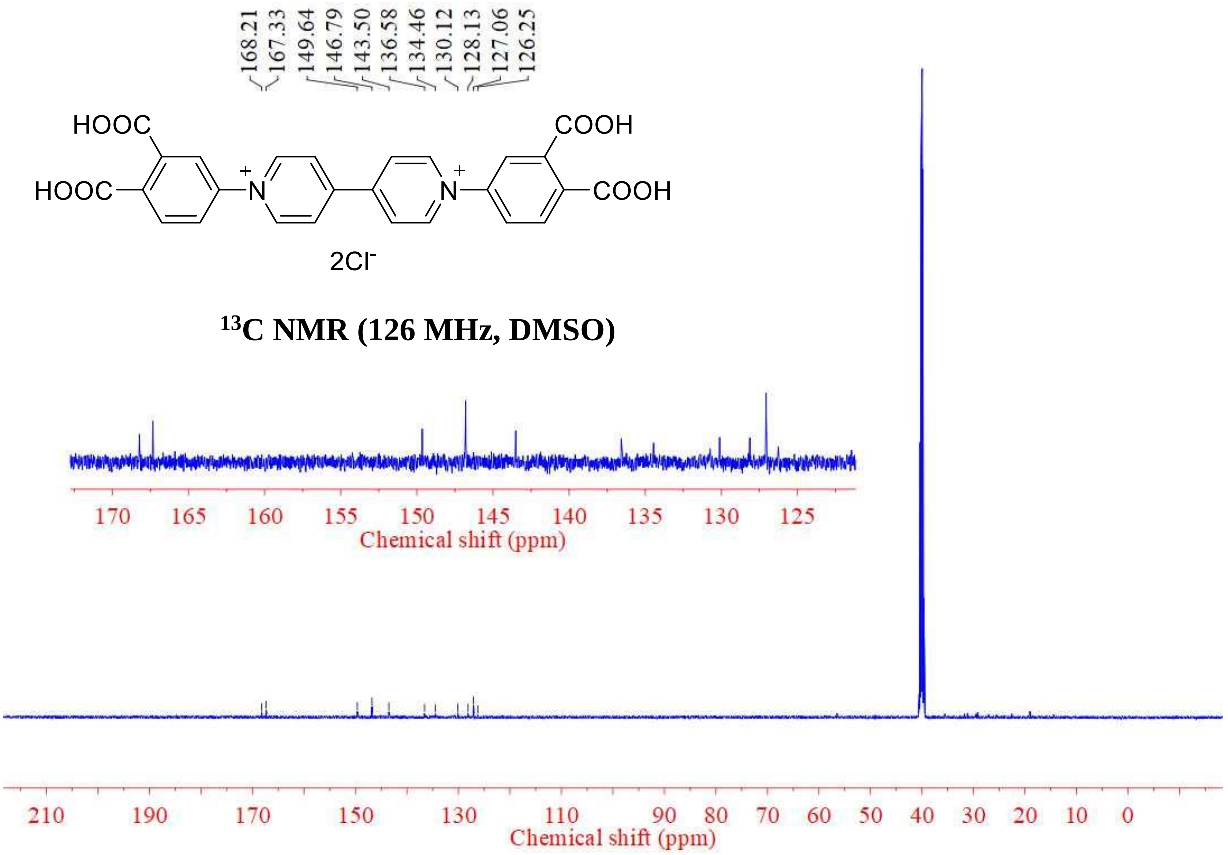

Supplementary Figure 16. ^{13}C-NMR spectrum of 1,1'-bis(3,4-dicarboxybenzyl)-4,4'-bipyridinium dichloride. ^{13}C NMR (126 MHz, DMSO) δ 168.21, 167.33, 149.64, 146.79, 143.50, 136.58, 134.46, 130.12, 128.13, 127.06, 126.25.

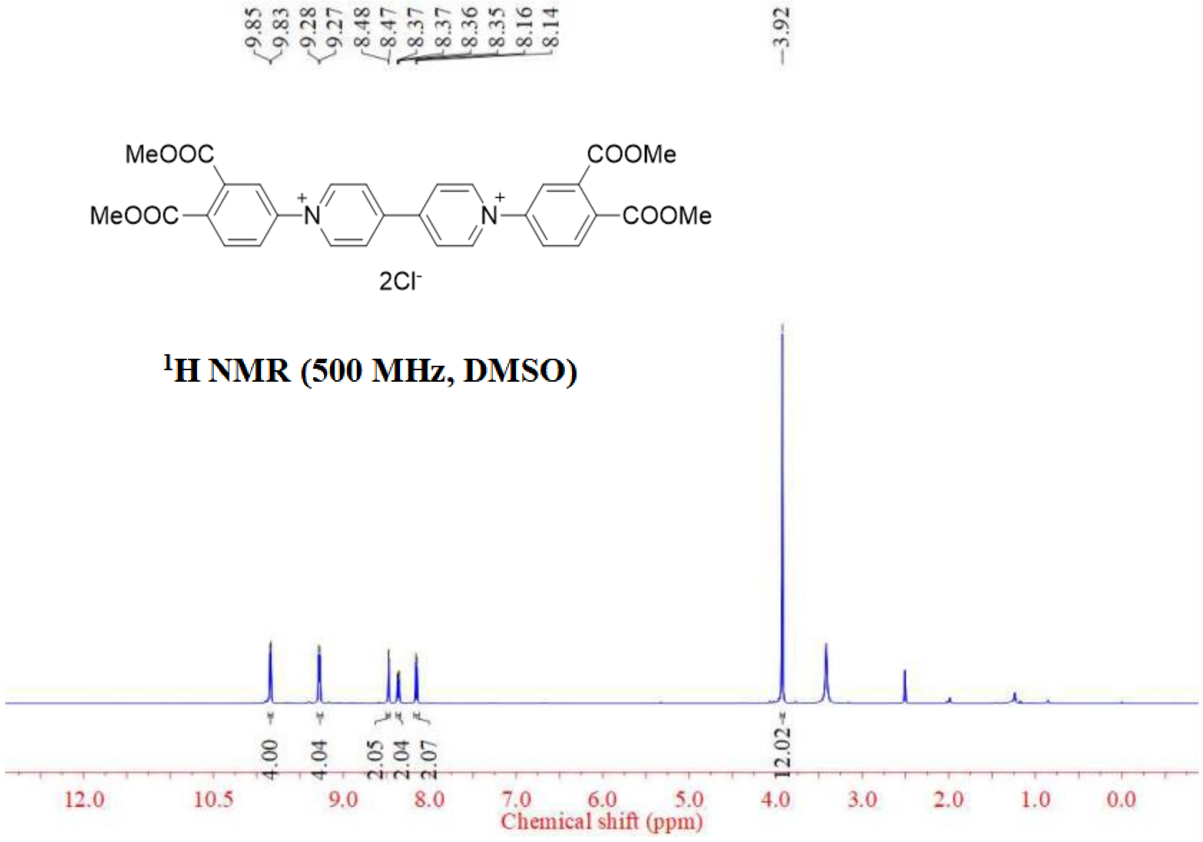

Supplementary Figure 17. ^{1}H-NMR spectrum of 1,1'-bis(3,4-dimethyl phthalate)-4,4'-bipyridinium dichloride. ^{1}H NMR (500 MHz, DMSO) δ 9.84 (d, *J* = 6.8 Hz, 4H), 9.28 (d, *J* = 6.9 Hz, 4H), 8.48 (d, *J* = 2.2 Hz, 2H), 8.36 (dd, *J* = 8.3, 2.3 Hz, 2H), 8.15 (d, *J* = 8.3 Hz, 2H), 3.92 (s, 12H).

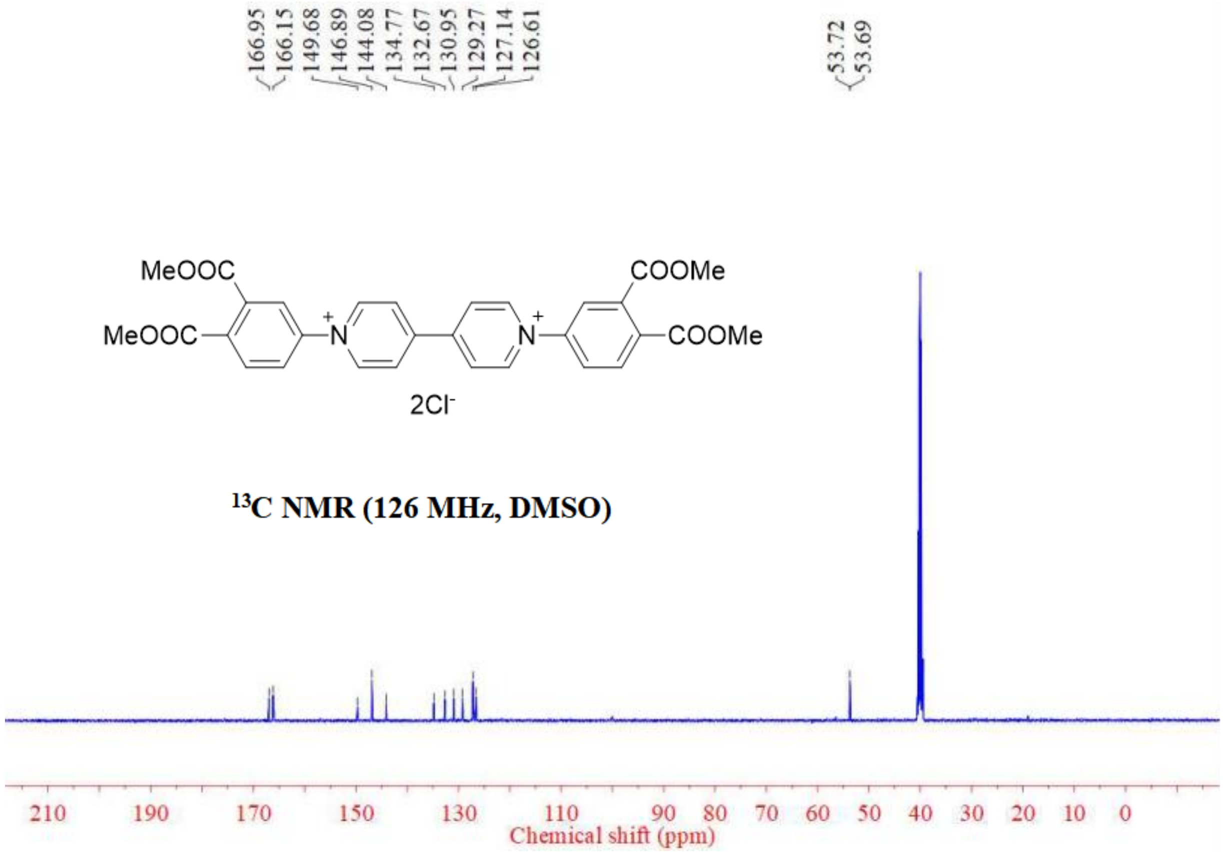

Supplementary Figure 18. ^{13}C-NMR spectrum of 1,1'-bis(3,4-dimethyl phthalate)-4,4'-bipyridinium dichloride.^{13}C NMR (126 MHz, DMSO) δ 166.95, 166.15, 149.68, 146.89, 144.08, 134.77, 132.67, 130.95, 129.27, 127.14, 126.61, 53.72, 53.69.

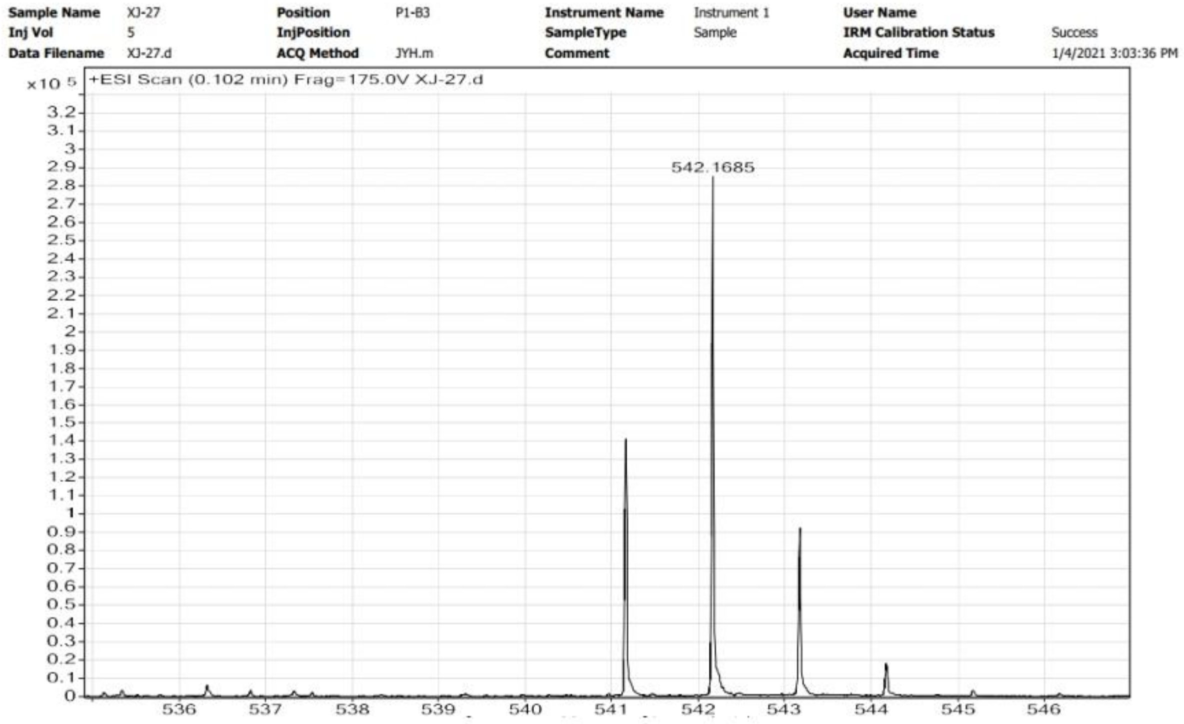

Formula (M)	Score (MFG)	Mass	Mass (MFG)	m/z (Calc)	Diff (ppm)	DBE	m/z
$C_{30}H_{26}N_2O_8^{2+}$	99.68	542.1685	542.1689	542.1689	0.77	19	542.1685

Supplementary Figure 19. High-resolution mass spectrometry for 1,1'-bis(3,4-dimethyl phthalate)-4,4'-bipyridinium dichloride.

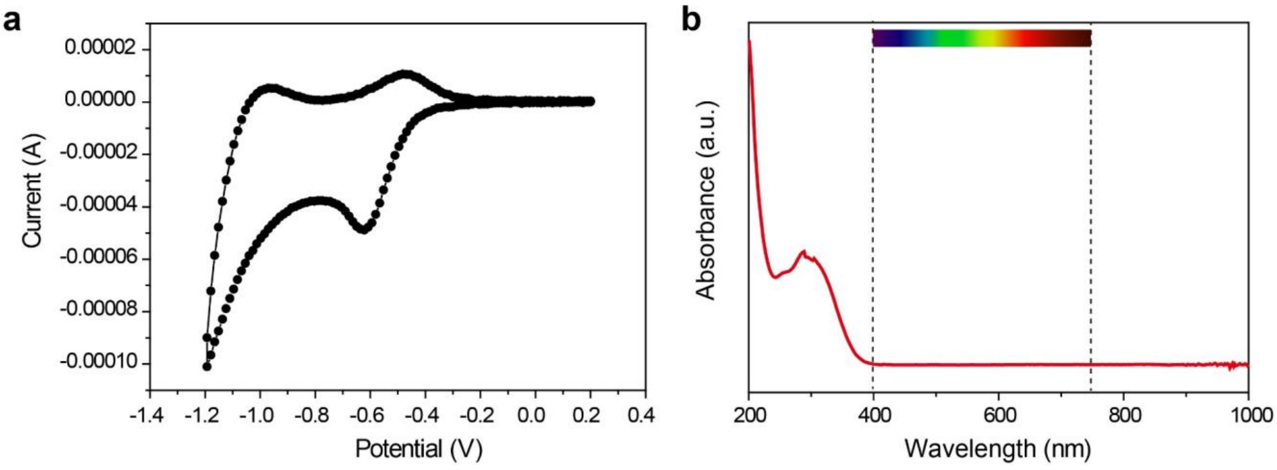

Supplementary Figure 20. a, Cyclic voltammogram of 1,1'-bis(3,4-dicarboxybenzyl)-4,4'-bipyridinium dichloride-modified, nanostructured TiO_2 electrode versus Ag/AgCl (1.0M KCl) reference electrode in an electrolyte consisting $LiClO_4$ (0.5 M) in propylene carbonate, indicating the presence of intermediates in redox reactions and the good reversibility of the reaction. **b,** Absorption spectrum of 1,1'-bis(3,4-dicarboxybenzyl)-4,4'-bipyridinium dichloride molecule at oxidized state as recorded in an aqueous solution.

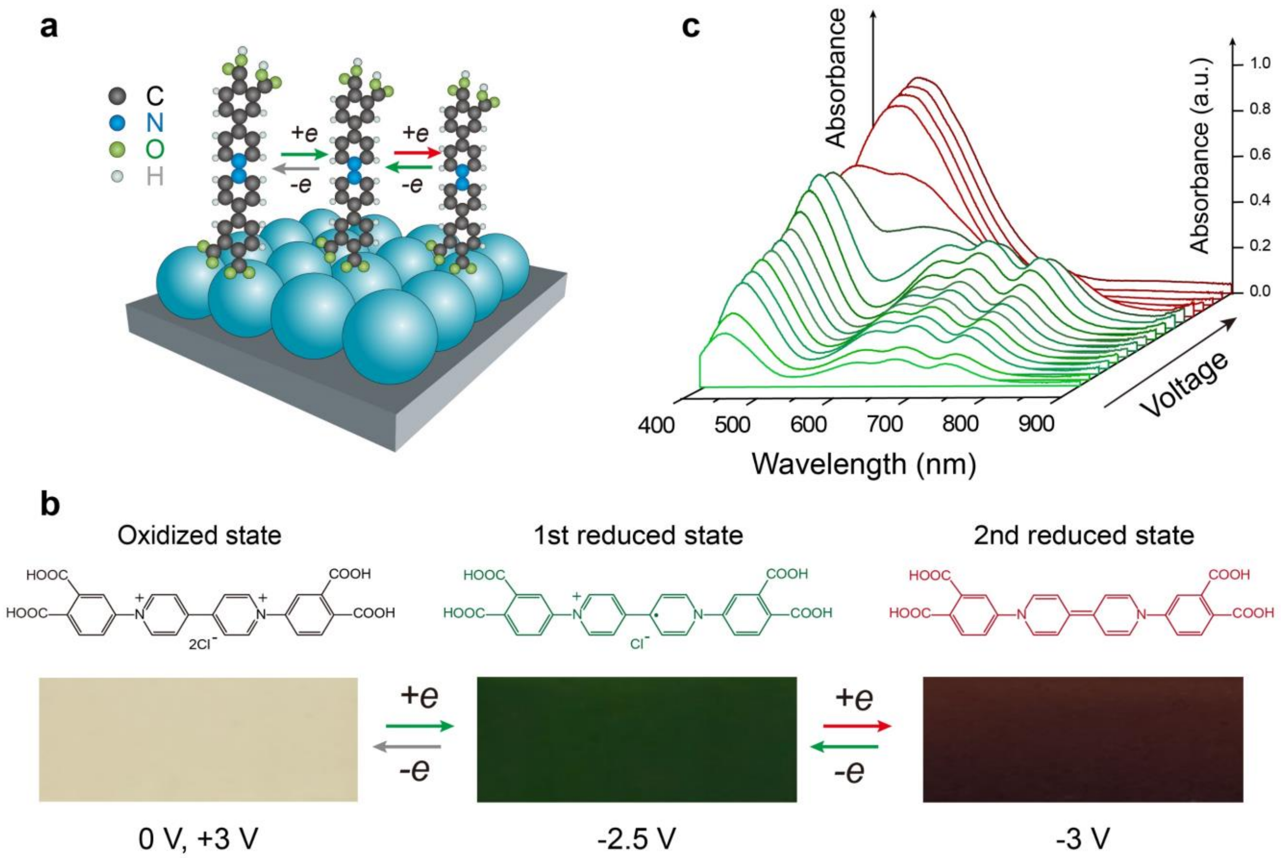

Supplementary Figure 21. a, Schematic of reversible electrochemical conversion of 1,1'-bis(3,4-dicarboxybenzyl)-4,4'-bipyridinium dichloride (CV) molecules. **b**, Chemical structures of the oxidized, first reduced, and second reduced states of the CV molecule and corresponding optical images of the CV-modified nanoparticle film under different applied potentials. **c**, Potential-dependent optical absorption spectra of CV molecules tethered on nanoparticle surfaces.

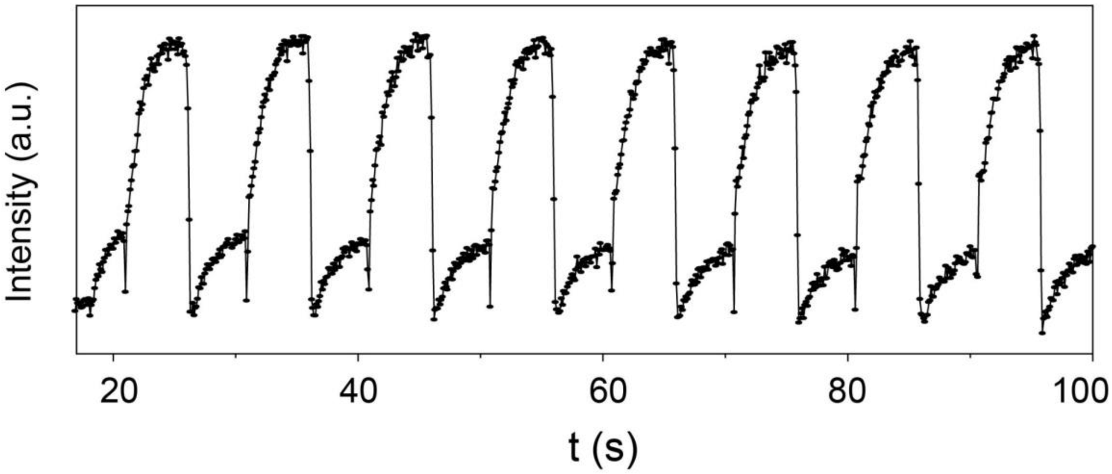

Supplementary Figure 22. Reversible luminescence on/off cycles, recorded at 654 nm by applying alternating applied potentials of ± 3.0 V to the Er-UCNP/CV based electrochemical cell. The Er-UCNP/CV based electrochemical cell exhibited excellent durability and fast switching speeds with rise time and fall delay on millisecond timescales.

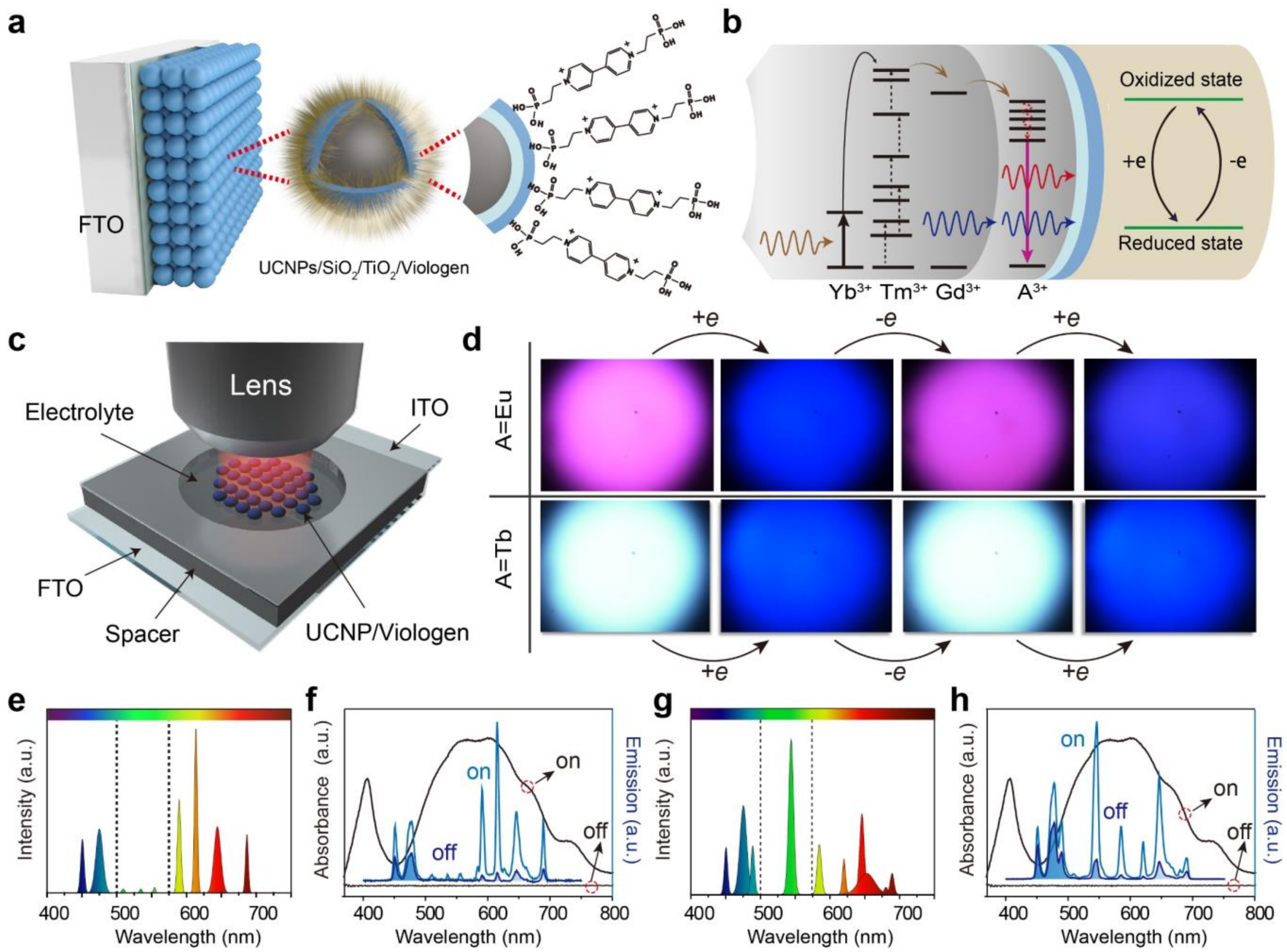

Supplementary Figure 23. a, Schematic of the electrochemical working electrode based on PV molecule-modified UCNPs. **b**, Proposed mechanism underlying the opto-electrochemical modulation of upconversion luminescence. **c**, Schematic of the experimental setup used to measure the photoluminescence characteristics of various samples. A 980-nm laser is focused through an objective to excite the hybrid film. **d**, Photoluminescence images of the hybrid device based on TiO_2-coated $NaGdF_4$:Yb/Tm@$NaYF_4$:Eu nanoparticles (Eu-UCNPs) (up) and $NaGdF_4$:Yb/Tm@$NaYF_4$: Tb nanoparticles (Tb-UCNPs) (bottom) under different applied potentials. **e**, Deconvolution of the emission spectrum of the Eu-UCNP-containing hybrid film into individual Gaussian peaks. **f**, Absorption spectra (black) of PV molecules anchored on the nanoparticle film and emission spectra (blue) of the hybrid film containing PV-modified Eu-UCNPs at on and off states. **g**, Deconvolution of the emission spectrum of Tb-UCNPs-containing hybrid film into individual Gaussian peaks. **h**, Absorption spectra (black) of CV molecule tethered on nanoparticle film and emission spectra (blue) of the Tb-UCNPs-containing hybrid film, recorded at on and off states.

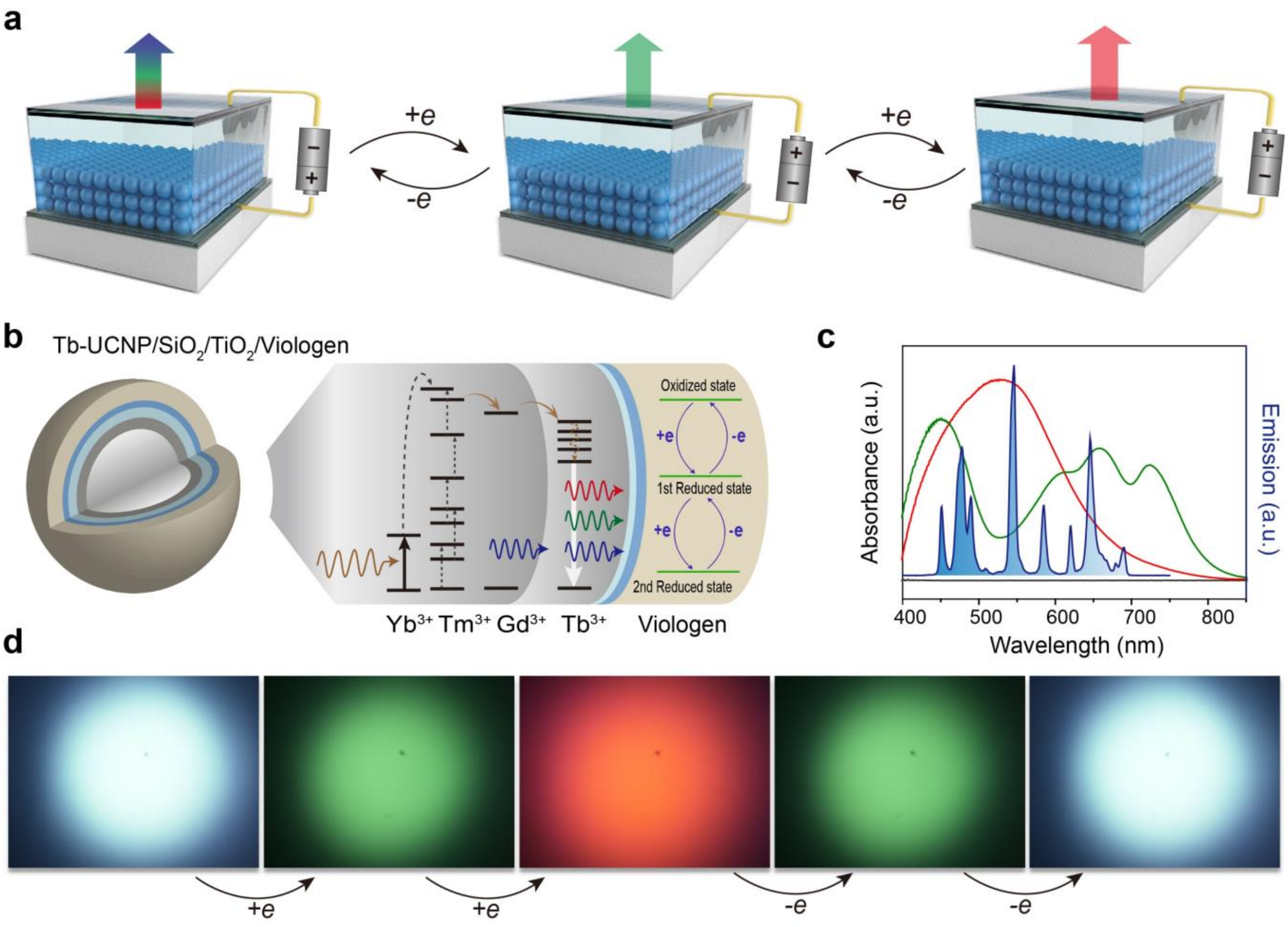

Supplementary Figure 24. **a**, Schematic illustration of upconversion multicolour switching of an electrochemical cell based on CV molecule-modified Tb-UCNPs. The Tb-UCNP/CV based electrochemical cell exhibited tunable upconversion luminescence under different potentials. **b**, Proposed mechanism underlying the opto-electrochemical modulation of upconversion luminescence. **c**, Emission spectrum (blue) of Tb-UCNPs-containing hybrid film and absorption spectra of CV molecules anchored on the nanoparticle film at oxidized (black), first reduced (green) and second reduced (red) states under different potentials. **d**, Photoluminescence images of Tb-UCNPs/CV based electrochemical cell under different potentials.

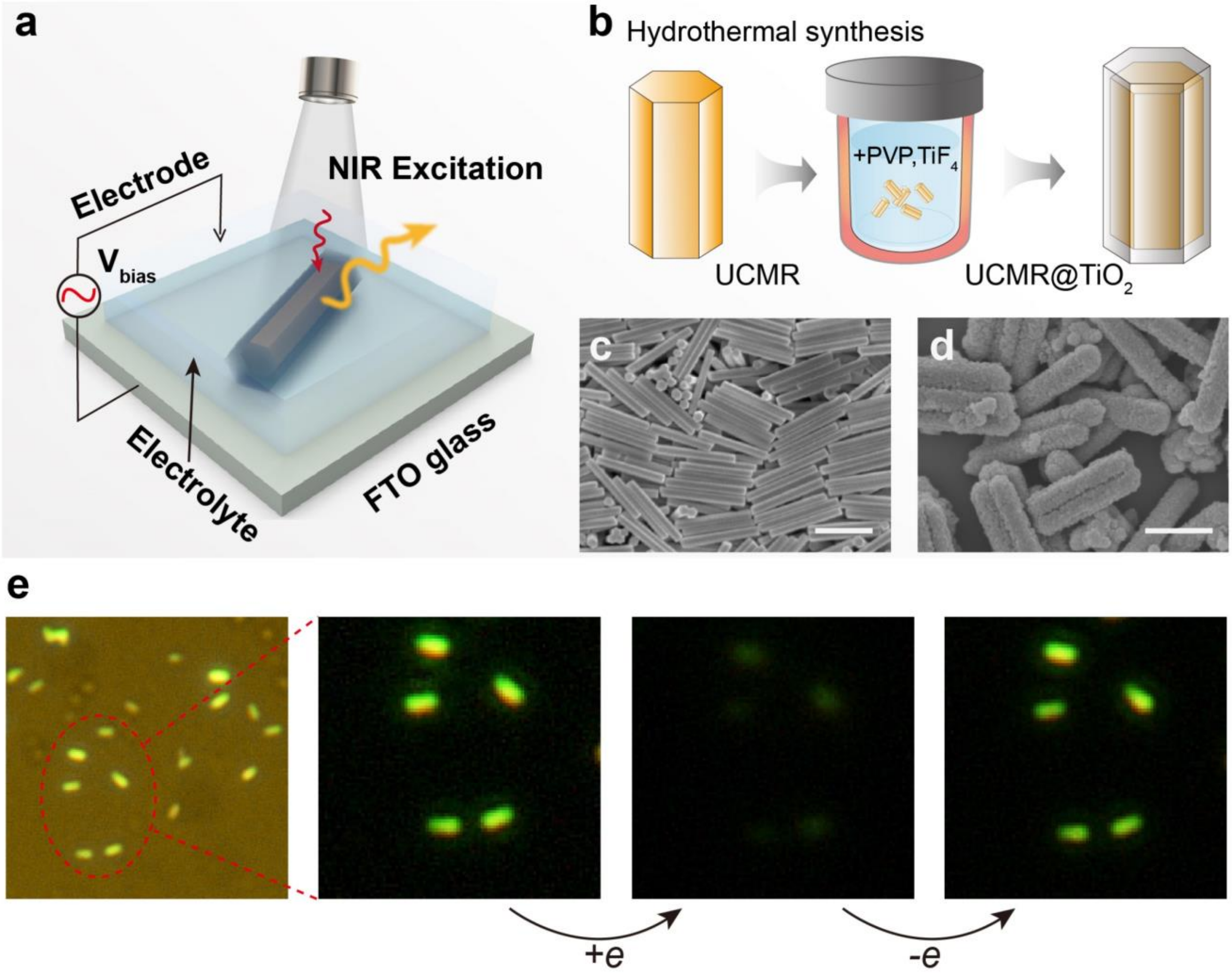

Supplementary Figure 25. a, Schematic of an electrochemical cell based on a single viologen molecule-modified $NaYF_4$:Yb/Er (18/2 mol%) upconversion microrod (UCMR). **b**, Schematic illustration of hydrothermal coating of TiO_2 onto upconversion microrods. **c,d,** SEM images of UCMRs and UCMR@TiO_2 core-shell structures, respectively. Scale bar: 1μm. **e**, Bright-field image and photoluminescence images of the opto-electrochemical device based on PV molecule-modified single microrods under different applied potentials, showing electrically switchable upconversion luminescence.

Table S1. The calculated maximum absorption wavelength ($\lambda_{abs.}$) and oscillator strength (f) of PV and CV molecules. The electronic transitions with high contributions are highlighted in red.

**Compound**	**$\lambda_{abs.}$ (nm)**	***f***	**Assignment**		
**PV (Cl = 1)**	387	0.06	α-HOMO-3	→	α-LUMO (9.6%)
			α-HOMO-3	→	α-LUMO+1 (3.7%)
			α-HOMO-2	→	α-LUMO (1.3%)
			α-HOMO-2	→	α-LUMO+1 (8.5%)
			α-HOMO	→	α-LUMO+1 (1.5%)
			β-HOMO-3	→	β-LUMO (2.0%)
			β-HOMO-2	→	β-LUMO+1 (42.4%)
			β-HOMO-2	→	β-LUMO+2 (1.7%)
			β-HOMO-2	→	β-LUMO+3 (1.9%)
			β-HOMO-1	→	β-LUMO+1 (1.6%)
			β-HOMO	→	β-LUMO+2 (2.0%)
			β-HOMO	→	β-LUMO+3 (1.3%)
	521	0.2	α-HOMO-3	→	α-LUMO+1 (1.5%)
			α-HOMO	→	α-LUMO (53.4%)
			α-HOMO	→	α-LUMO+1 (27.1%)
			α-HOMO	→	α-LUMO+2 (2.7%)
			β-HOMO-3	→	β-LUMO (5.0%)
			β-HOMO	→	β-LUMO+1 (1.3%)
			β-HOMO	→	β-LUMO+2 (5.8%)
	645	0.0012	α-HOMO-3	→	α-LUMO (1.4%)
			α-HOMO-3	→	α-LUMO+1 (2.8%)
			α-HOMO	→	α-LUMO (32.4%)
			α-HOMO	→	α-LUMO+1 (59.7%)
			α-HOMO	→	α-LUMO+2 (1.2%)
**PV (Cl = 2)**	272	0.03	HOMO-3	→	LUMO+2 (30.8%)
			HOMO-3	→	LUMO+3 (50.7%)
			HOMO-2	→	LUMO+3 (6.2%)
			HOMO	→	LUMO+3 (5.8%)
**CV (Cl = 0)**	509	0.15	HOMO	→	LUMO (4.5%)
			HOMO	→	LUMO+1 (11.0%)
			HOMO	→	LUMO+2 (77.5%)
			HOMO	→	LUMO+3 (2.3%)
			HOMO	→	LUMO+4 (4.2%)
**CV (Cl = 1)**	476	0.04	α-HOMO-3	→	α-LUMO (45.6%)
			α-HOMO-3	→	α-LUMO+1 (6.7%)
			α-HOMO	→	α-LUMO+3 (5.7%)
			β-HOMO	→	β-LUMO+1 (9.3%)
			β-HOMO	→	β-LUMO+2 (24.5%)

			β-HOMO	→	β-LUMO+3 (1.4%)
	568	0.12	α-HOMO-3	→	α-LUMO (5.2%)
			α-HOMO	→	α-LUMO (5.1%)
			α-HOMO	→	α-LUMO+1 (3.3%)
			α-HOMO	→	α-LUMO+4 (5.1%)
			β-HOMO	→	β-LUMO (1.8%)
			β-HOMO	→	β-LUMO+1 (71.7%)
			β-HOMO	→	β-LUMO+2 (3.7%)
	702	0.45	α-HOMO-3	→	α-LUMO (1.0%)
			α-HOMO	→	α-LUMO (84.6%)
			α-HOMO	→	α-LUMO+1 (1.2%)
			β-HOMO-3	→	β-LUMO (1.0%)
			β-HOMO	→	β-LUMO (5.0%)
			β-HOMO	→	β-LUMO+1 (5.0%)
**CV (Cl = 2)**	324	0.04	HOMO-3	→	LUMO+2 (11.5%)
			HOMO-1	→	LUMO+2 (15.5%)
			HOMO-1	→	LUMO+3 (23.1%)
			HOMO	→	LUMO+3 (28.2%)
			HOMO	→	LUMO+4 (9.3%)
			HOMO	→	LUMO+5 (7.7%)

References

1. Frisch, M.; Trucks, G.; Schlegel, H.; Scuseria, G.; Robb, M.; Cheeseman, J.; Scalmani, G.; Barone, V.; Petersson, G. & Nakatsuji, H. *Inc., Wallingford CT* 2016.

2. Dennington, R.; Keith, T. & Millam, J. *Semichem Inc.: Shawnee Mission, KS* 2009.
